# Alternative microexon splicing code for a four-amino acid peptide of PTPRD governs behavioral development

**DOI:** 10.1073/pnas.2515310123

**Published:** 2026-04-08

**Authors:** Ayako Imai, Hironori Izumi, Nagomi Ito, Hina Ogiso, Yuki Kitajima, Shuhei Kawase, Mizuki Sendo, Kenji Azechi, Toshihide Tabata, Yumie Koshidaka, Shuya Fukai, Keizo Takao, Hisashi Mori, Tomoyuki Yoshida

**Affiliations:** ^a^Department of Molecular Neuroscience, Graduate School of Medicine and Pharmaceutical Sciences, University of Toyama, Toyama 930-0194, Japan; ^b^Research Center for Idling Brain Science, University of Toyama, Toyama 930-0194, Japan; ^c^Department of Veterinary Nursing and Health Science, School of Veterinary Medicine, Azabu University, Kanagawa 252-5201, Japan; ^d^Department of Molecular Neuroscience, Interdisciplinary Graduate School of Medicine, Pharmacy, Sciences and Engineering, University of Toyama, Toyama 930-0194, Japan; ^e^Department of Biofunctional Molecular Chemistry, Faculty of Engineering, University of Toyama, Toyama 930-8555, Japan; ^f^Department of Biological Information Processing, Graduate School of Science and Engineering, University of Toyama, Toyama 930-8555, Japan; ^g^Life Science Research Center, University of Toyama, Toyama 930-0194, Japan; ^h^Department of Chemistry, Graduate School of Science, Kyoto University, Kyoto 606-8502, Japan

**Keywords:** microexon, alternative splicing, PTPRD, synapse organizer, behavior

## Abstract

Microexons of 3 to 27 nucleotides selectively regulated in the vertebrate nervous system have attracted attention as new elements for modifying the function of neuronal proteins. Protein tyrosine phosphatase δ (PTPRD) is one of presynaptic hubs for neuronal synaptic organization. Alternative splicing (AS) of three microexons, meA3, meA6, and meB, encoding 3, 6, and 4 amino acid-peptides, respectively, imparts structural diversity to PTPRD, due to which the resulting eight splice variants exhibit distinct synaptogenic properties. However, the regulatory mechanisms of AS and physiological significance of the AS code for *Ptprd* gene remain largely unknown. Here, we report the AS of the three microexons is genetically regulated to generate spatiotemporally distinct expression pattern of eight *Ptprd* splice variants across brain regions and is modulated by neuronal activity. We identified both the intronic splicing enhancer region (ISE) for meB contributing to the spatiotemporal patterning of the AS and the neuronal activity–dependent intronic splicing silencer region for meB (ISS). Heterozygous deletion of the ISE in mice led to a decreased meB selection rate by ~25% with unaltered total amount of PTPRD protein and caused severe sensory, motor, social, and emotional behavioral abnormalities but no obvious changes in learning and memory, while heterozygous *Ptprd* knockout mice with unaltered meB selection rate and ~50% decrease in total PTPRD protein showed much fewer behavioral abnormalities. Interestingly, deletion of the ISS for meB caused selective impairments in motor learning and fear memory. Our findings demonstrate the spatiotemporal and activity-dependent AS code for *Ptprd* meB plays a crucial role in proper behavioral development.

## Significance

How animal behavior develops is still a mystery. It is presumably determined to some extent by genes and modulated by environment and experience. We here find both genetic and environment-dependent splicing regulatory elements for the microexon encoding a mere four-amino acid-peptide of Protein tyrosine phosphatase δ (PTPRD), a major neuronal synapse organizer. Manipulation of the genetic regulatory element to decrease the microexon selection rate by ~25% in mice causes severe defects in a broad range of behavioral domains including sensory-motor functions, emotion, motivation, and sociability. In contrast, lack of the environment-dependent regulatory element leads to selective impairments in learning and memory. This study highlights the crucial role of the alternative splicing code of a single microexon for genetic and environment-dependent behavioral development.

The genome, the original blueprints of life, can give rise to various transcripts via alternative splicing (AS) of precursor mRNAs resulting in transcriptomic and proteomic complexity and diversity. AS occurs more frequently in higher eukaryotes, thus it is considered a key machinery for diversification and evolution (1, 2). Among all human multiexon genes, 95% undergo AS, and the resulting splice variants are variably expressed in different cells and tissue-types (3–5). Neurons have developed particular modes of AS to regulate their highly specialized structures and functions (6). Microexons, consisting of 3 to 51 nucleotides (nts) and typically multiples of three nts in length, are highly utilized in neuronal cell lineage and play a crucial role in neuronal development and differentiation (7, 8). Although microexons represent only ~1% of all AS events, they comprise one-third of all neural-regulated AS events conserved between human and mouse (7). Moreover, the misregulation of microexons is implicated in neurodevelopmental disorders, including autism spectrum disorder (ASD) (6, 7).

Protein tyrosine phosphatase δ (PTPRD) is a presynaptic hub protein belonging to type IIA receptor protein tyrosine phosphatases, which induce synapse formation through trans-synaptic interactions with selective postsynaptic binding partners (9, 10). PTPRD exists as at least eight extracellular-domain splice variants generated by the inclusion or exclusion of three microexons: meA3, meA6, and meB, which encode 3-, 6-, and 4-amino acid peptides, respectively (11). The meA3- and meA6-derived peptides are inserted into a loop within the extracellular second immunoglobulin (Ig)-like domain, whereas meB-derived peptide at the junction between the second and third Ig-like domains, to regulate their configuration and the structures of the binding interfaces to the postsynaptic binding partners, thereby giving distinct synaptogenic properties to the eight splice variants (12–16). In the cultured cerebral cortical neurons, the length of meA peptides determines synaptogenic activity, i.e., the longer meA peptides, the greater the synaptogenic activity (16). In contrast, the choice of meB peptide is crucial for determining synaptic properties. PTPRD variants containing the meB peptide selectively induce excitatory synapses through interleukin-1 receptor accessory protein (IL-1RAcP), IL-1RAcP-like 1 (IL1RAPL1), Slitrks, and SALMs, whereas those lacking the meB peptide are involved in excitatory and inhibitory synaptic differentiation through neuroligin-3 (NLGN3) (10, 16). Importantly, most of the PTPRD splice-variant-specific ligands are associated with neurodevelopmental and psychiatric disorders (9, 17). Furthermore, *Nlgn3* mutation that selectively lack the PTPRD splice variant-specific interaction and *Ptprd* splice variant-specific knockout cause neurodevelopmental disorder-related behavioral phenotypes (16, 18–20). However, the AS regulatory mechanisms, and physiological significance of the AS program of these *Ptprd* microexons in vivo remain largely unknown.

In this study, we demonstrated the different contribution of meA- and meB-derived peptides for synaptogenic properties in cultured neurons from the different brain areas. Spatiotemporal mapping of the eight splice variants of *Ptprd* in the brain revealed the region- and age-specific distribution of these variants via genetic AS programs. Furthermore, we found that high-potassium-induced activation of cultured neurons induced meB skipping. Genome-edited mice lacking intronic sequences containing consensus splicing enhancers (ISEs) to decrease the meB selection rate exhibited severe abnormalities in sensory, motor, and emotional behaviors, with unaltered learning ability and memory. In contrast, those lacking the neuronal activity–dependent intronic splicing silencer (ISS) responsible for meB skipping showed selective impairments in learning and memory. Our results demonstrate that AS programs for the inclusion or exclusion of a mere 4-amino acid meB peptide of PTPRD govern proper behavioral development.

## Results

### meA- and meB-Derived Peptides Differentially Contribute to Synaptogenic Property of Eight PTPRD Splice Variants in Different Neuronal Culture Systems.

**Fig. 1.**
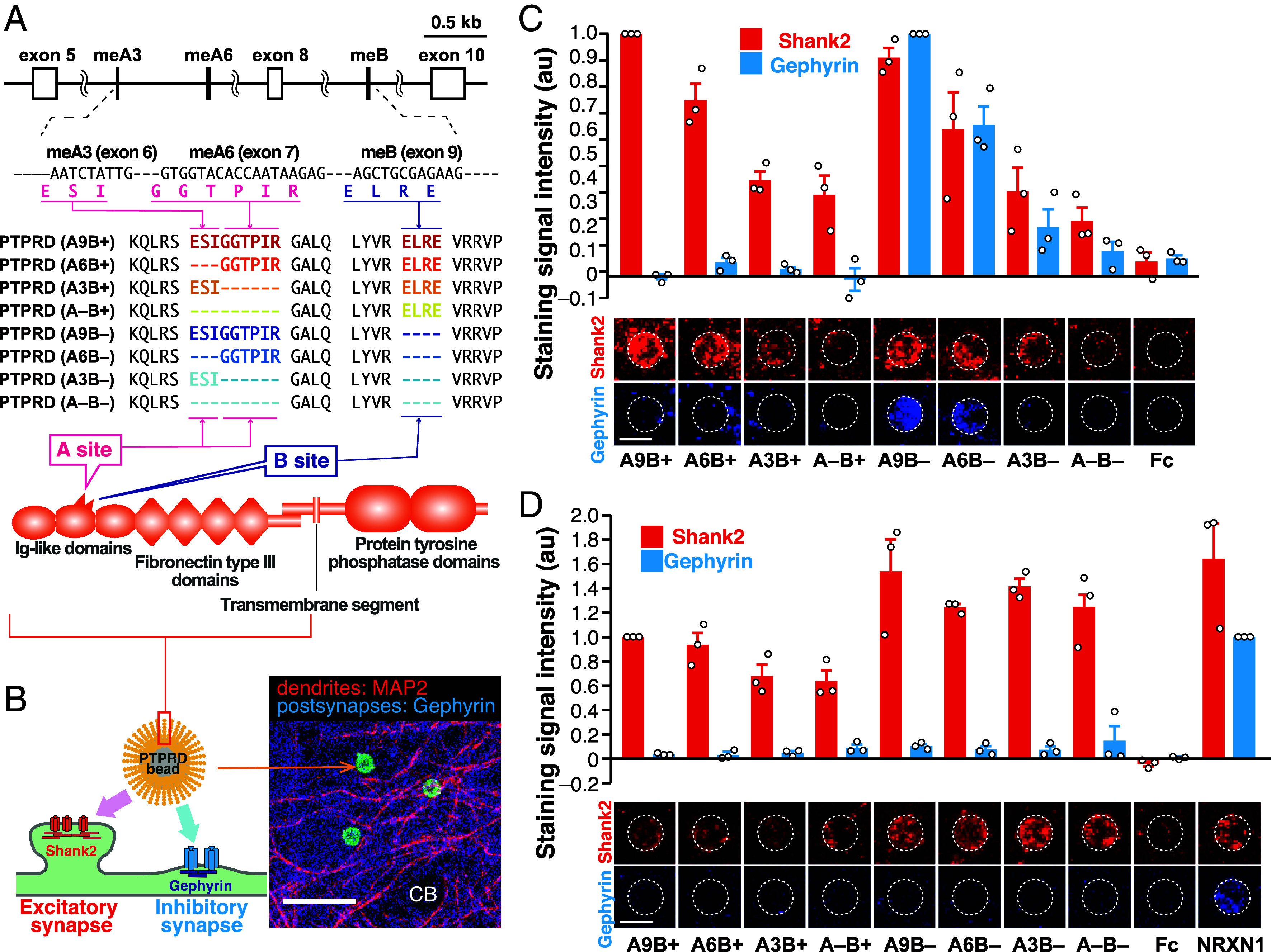
Synaptogenic property of eight PTPRD splice variants. (*A*) Schematic exon–intron structure of *Ptprd* gene and amino acid sequences of splice variants in Ig-like domains of PTPRD protein. (*B*) Schema for hemisynapse formation assay between PTPRD ECD-conjugated beads and cultured neurons. Representative low magnification image of neuron-bead coculture immunostained for MAP2 and Gephyrin is shown on the *Right*. CB, neuronal cell body. (Scale bar, 20 μm.) (*C* and *D*) Induction of postsynaptic differentiation of cultured hippocampal (*C*) and cerebellar (*D*) neurons by beads conjugated with ECDs of PTPRD splice variants. Excitatory and inhibitory postsynaptic terminals were visualized by immunostaining for Shank2 (red) and gephyrin (blue), respectively (*Bottom*). Shank2 (red bars) and gephyrin (blue bars) staining signals on the beads were quantified (*Top*). (Scale bar, 5 μm.) The dashed circles indicate the positions of the beads. Data are presented as mean ± SEM. (n = 3 experiments, each).

*Ptprd* meA3-, meA6-, and meB-derived peptides are alternatively inserted at the interfaces between the Ig-like domains of PTPRD and postsynaptic binding partners, resulting in eight PTPRD splice variants (Fig. 1*A*) (12–16). We previously reported the length of meA peptides contributed to the synapse-inducing activity whereas choice of meB peptide determined synaptic property, i.e., excitatory or inhibitory, in the cultured cortical neurons (16). However, it is not known yet if the same is true in the cultured neurons from different brain regions. To study the synaptogenic properties further we applied the beads coated with the eight PTPRD splice variants onto cultured cerebral cortical, hippocampal, and cerebellar neurons. After incubation for 24 h, we detected punctate staining signals for Shank2 and gephyrin, the marker for excitatory and inhibitory postsynaptic terminals, respectively, on the contacting dendrites of the cultured neurons (Fig. 1*B*). In the hippocampal neurons, the length of meA peptides determined the synaptogenic activity while meB determined the synaptic properties as it is seen in the cortical neurons (Fig. 1*C* and *SI Appendix*, Fig. S1) (16). Interestingly, it was not meA3 and meA6 peptides but meB peptide that regulates synaptogenic activity in the cerebellar neurons (Fig. 1*D*). These results suggest that the meA and meB peptides contribute differentially to synaptogenic properties in different brain regions.

### Spatiotemporal and Activity-Dependent AS Regulation of *Ptprd* Microexons.

**Fig. 2.**
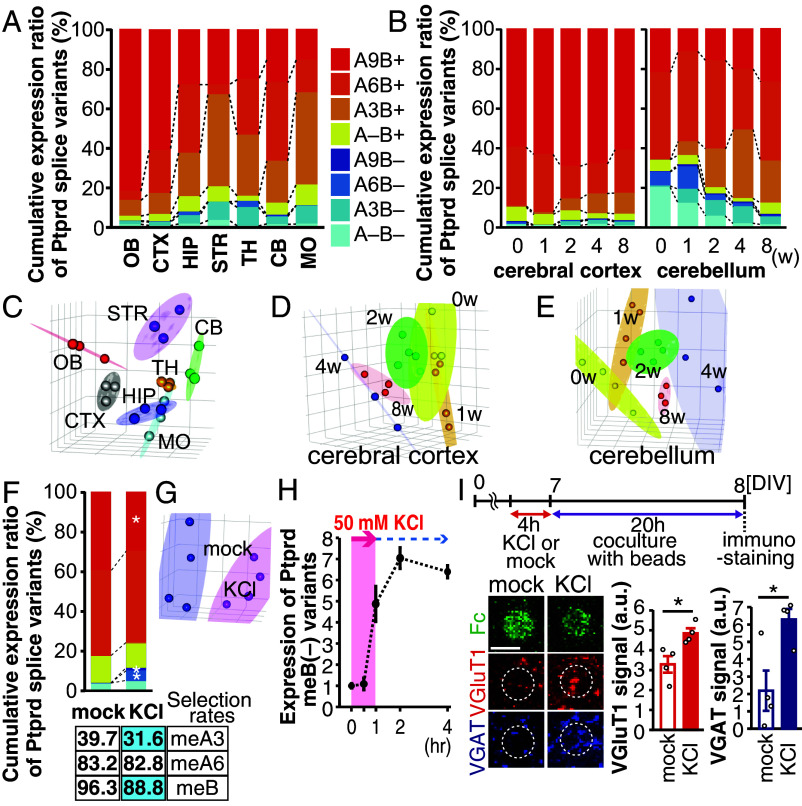
Spatiotemporal expression of eight *Ptprd* splice variants. (*A*) Relative abundance ratios of eight *Ptprd* splice variants in the OB, CTX, HIP, STR, TH, CB, and MO of 8-wk-old mice (N = 3). (*B*) Developmental changes in relative abundance ratios of eight *Ptprd* splice variants in the CTX and CB (N = 3). (*C*) PCoA on the data shown in (*A*). (*D* and *E*) PCoA on the data shown in (*B*). (*F*) Relative abundance ratios of eight *Ptprd* splice variants (*Top*) and selection rates of meA3, meA6, and meB (*Bottom*) in the mock- and KCl-treated primary cerebral cortical neurons (n = 4 experiments, each). Statistically significant differences in two-tailed *t* test are shaded in light blue. (*G*) PCoA on the data shown in (*F*). (*H*) Time course of *Ptprd* meB skipping after KCl treatment measured by quantitative real time PCR for *Ptprd* meB(–) variants (n = 3 experiments for each time point). (*I*) Increase in PTPRD meB(–) variants-mediated synaptogenic activity after KCl treatment. Experimental design (*Top*), representative images (*Bottom* *Left*), and quantitative measurements (*Bottom* *Right*) of presynaptic differentiation of cultured cortical neurons by NLGN3^HSE^-Fc beads. Excitatory and inhibitory presynaptic terminals were visualized by immunostaining for VGluT1 (red) and VGAT (blue), respectively. (Scale bar, 5 μm.) The dashed circles indicate the positions of the beads. Data are presented as mean ± SEM. (n = 4 experiments).

As brain regions with specific functions comprise characteristic neurons with specialized synaptic connections and properties, we examined the spatiotemporal distribution pattern of the eight *Ptprd* splice variants in different brain regions of mice, including the olfactory bulb (OB), cerebral cortex (CTX), hippocampus (HIP), striatum (STR), thalamus (TH), cerebellum (CB), and medulla oblongata (MO). The relative abundances of these variants were determined using a cDNA fingerprinting technique with the amplified *Ptprd* fragments (*SI Appendix*, Fig. S2). The results revealed the brain region-specific distribution of these splice variants (Fig. 2*A*). Principal coordinate analysis (PCoA) of the relative abundance of eight *Ptprd* splice variants in each mouse confirmed brain region specificity with small interindividual variation (Fig. 2*C*). Furthermore, temporal analysis at 0, 1, 2, 4, and 8 wk of postnatal age revealed the age-specific distribution of the relative abundance of the eight *Ptprd* splice variants in the CTX and CB (Fig. 2*B*). PCoA confirmed small interindividual variation within the same age group (Fig. 2 *D* and *E*).

Synapse formation is genetically programmed but can be altered based on neuronal activity (21, 22). Therefore, neuronal activity may affect the AS of *Ptprd* microexons. In mouse primary cerebral cortical neurons, the *Ptprd* splice variants lacking meB markedly increased between days in vitro (DIV) 8 and DIV16 (*SI Appendix*, Fig. S3). Because this culture system already had high synaptogenic activity by DIV8 (*SI Appendix*, Fig. S3), it was thought that neural activity might cause changes in the expression of the meB-lacking splice variants. To verify this, mouse primary cerebral cortical neurons at DIV7 were stimulated with 50 mM potassium chloride (KCl) for 4 h, which strongly induced expression of neuronal activity–dependent genes, *Fos* and *Bdnf* (*SI Appendix*, Fig. S4). We found the relative abundance of the eight *Ptprd* splice variants was indeed altered after KCl treatment. The expression of the *Ptprd* A9B+ variant was decreased, whereas that of the *Ptprd* A9B– and *Ptprd* A6B– variants was increased (Fig. 2*F*). The selection rates of each microexon calculated based on the relative abundance of the eight *Ptprd* splice variants suggest that neuronal activity induces meA3 and meB skipping (Fig. 2*F*). The neuronal activity–induced meB skipping seemed to be a very rapid response, as it was observed as early as 1 h after KCl administration (Fig. 2*H*). In order to examine whether neuronal activity–induced meB skipping actually led to change in level of PTPRD proteins lacking meB peptide, coculture assay was performed using cortical neurons and beads conjugated with NLGN3^HSE^ mutant protein that induces presynaptic differentiation through the selective interactions with the meB-peptide-lacking PTPRD variants (Fig. 2*I*) (16). Increments in immunostaining signals for vesicular glutamate transporter 1 (VGluT1) and vesicular GABA transporter (VGAT) on the beads after KCl treatment suggest that the neuronal activity increases expression of the meB-peptide-lacking PTPRD variants and causes changes in synaptogenic property of the cortical neurons (Fig. 2*I*). Although these results imply that the AS of these three microexons of *Ptprd* gene is genetically and neuronal activity dependently regulated to generate spatiotemporal synaptogenic properties of neurons, the regulatory mechanisms and physiological roles of this AS program remain largely unknown.

### Upstream Intronic Splicing Enhancer Sequence for *Ptprd* meB.

**Fig. 3.**
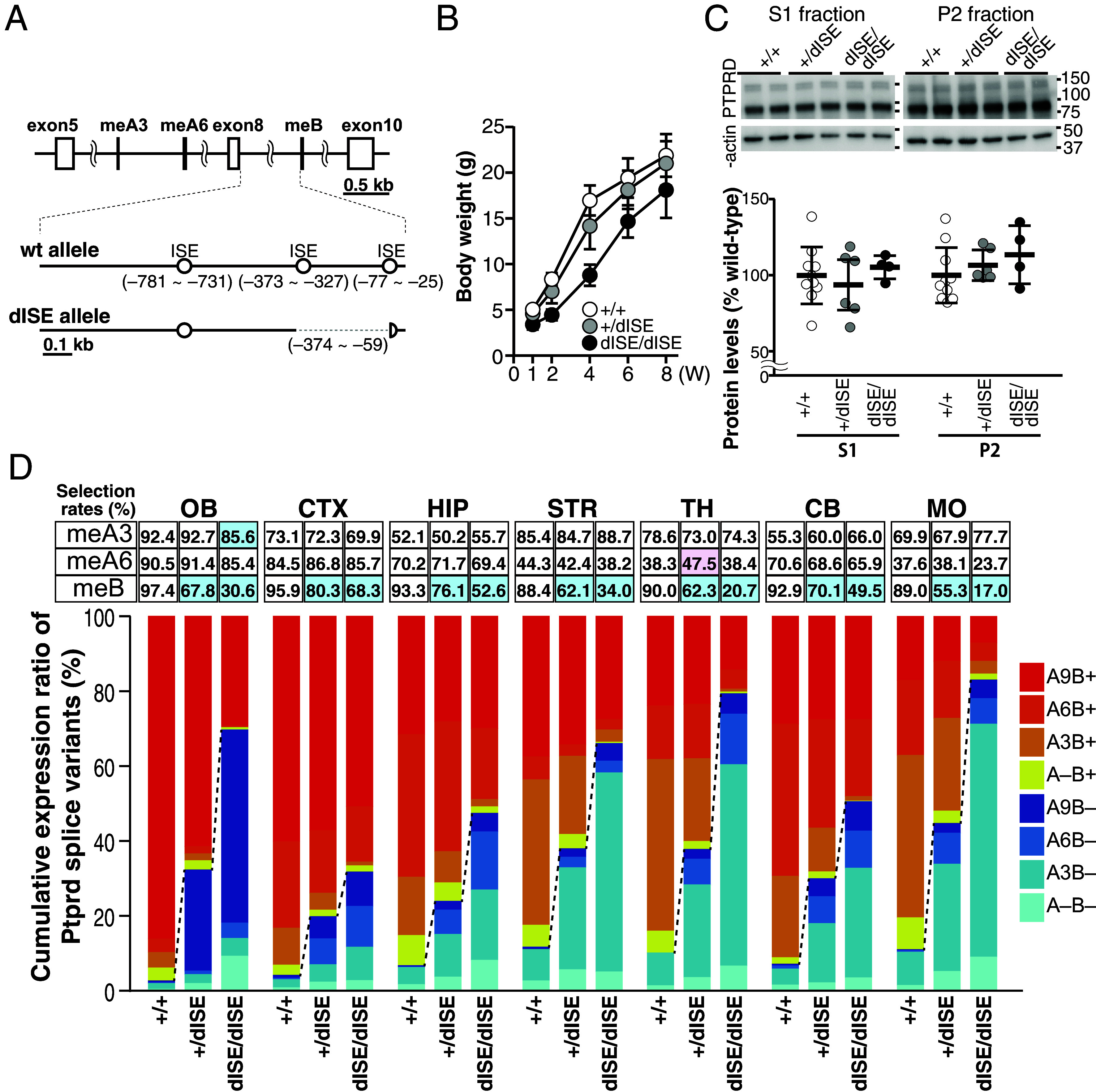
Intronic splicing enhancer region for *Ptprd* meB. (*A*) Schematic presentation of *Ptprd* exon–intron structure around meA3, meA6, and meB, and dISE mutant allele. (*B*) Developmental changes in body weights of homozygous (N = 6) and heterozygous (N = 19) dISE mutant mice and their WT littermates (N = 12). (*C*) Representative immunoblots (*Top*) and quantification of expression levels (*Bottom*) of total PTPRD protein in the whole brain S1 fraction and synaptosomal P2 fraction in *Ptprd^+/dISE^, Ptprd^dISE/dISE^*, and their littermate WT mice (N = 10, 6, and 4 mice, respectively). (*D*) Selection rates of meA3, meA6, and meB (*Top*) and relative abundance ratios of eight *Ptprd* splice variants (*Bottom*) in the OB, CTX, HIP, STR, TH, CB, and MO of 8-wk-old WT, heterozygous dISE mutant, and homozygous dISE mutant mice (N = 3, each). Statistically significant differences in Dunnett test comparison with WT are shaded in light blue or pink. Data are mean ± SEM for (*B*) and mean ± SD for (*C*).

Because the meB-derived peptide of PTPRD plays a crucial role in the regulation of synaptogenic properties in cultured neurons (Fig. 1 *C* and *D* and *SI Appendix*, Fig. S1) (16), we next focused on the regulatory mechanism and physiological significance of the AS code for meB. The intronic consensus UCUCU/UGC motifs that recruit Srsf11 and Srrm4 are known to function as ISEs for downstream microexons (23). In fact, the upstream intron of meB (intron 8) contains three such consensus ISE motifs (Fig. 3*A*). Because Srsf11 was shown to bind primarily exon-proximal intronic regions of neuronal microexons (23), we generated mutant mice lacking the 316-bp intronic sequence containing two of the three consensus ISE motifs for meB (*Ptprd^dISE^*, Fig. 3*A*). Indeed, in a minireporter gene assay for meB splicing, we confirmed that the meB selection rate was significantly reduced by deleting the 316-bp intronic sequence and the UCUCU motif closest to meB contributed to the meB selection (*SI Appendix*, Fig. S5). Approximately half of the homozygous mutants (9 out of 18) died within the first wk of life. The surviving mice showed significantly decreased body weight compared with the wild-type (WT) and heterozygous mutant mice, suggesting severe developmental delay (Fig. 3*B*). In contrast, the heterozygous mutant mice appeared to develop normally, as their body weight trend was comparable to that of the WT mice. Analysis of the spatial distribution patterns of the eight *Ptprd* splice variants across different brain regions including the OB, CTX, HIP, STR, TH, CB, and MO of mice with *Ptprd^dISE^* mutation at 8 wk of age revealed that the selection rates of meB decreased by ~16 to 34% and ~28 to 72% in *Ptprd^+/dISE^* and *Ptprd^dISE/dISE^* mice, respectively. In contrast, selection rates of meA3 and meA6 remained mostly unchanged (Fig. 3*D*). Despite the changes in transcript composition by the *Ptprd^dISE^* mutation, the levels of the total PTPRD protein in the whole brain, at least in cellular S1 and synaptosomal P2 fractions, were comparable among WT, heterozygous mutant, and homozygous mutant mice (Fig. 3*C*). These results suggest that the intronic 316-bp sequence functions as a splicing enhancer for meB, although the contribution of this sequence seems to depend on the brain region. Since changes in the selection rate of meB in our mutant mice were predicted to alter the synaptogenic properties of neurons, we tested this in cultured cortical neurons and cortical brain slices of WT and *Ptprd^+/dISE^* mice. In the primary cortical neurons from *Ptprd^+/dISE^* mice, IL1RAPL1 bead-mediated excitatory synapse formation (11) was down-regulated, while NLGN3^HSE^ bead-mediated inhibitory synapse formation (16) was up-regulated (*SI Appendix*, Fig. S6 *A* and *B*). Furthermore, immunostaining signal ratios for VGluT1 compared to VGAT in the CTX were lower in the *Ptprd^+/dISE^* mice than littermate WT mice, suggesting that decreased selection rate of *Ptprd* meB causes excitatory/inhibitory synaptic imbalance (*SI Appendix*, Fig. S6*C*).

### A Decreased meB Selection Rate Causes Severe Behavioral Abnormality.

**Fig. 4.**
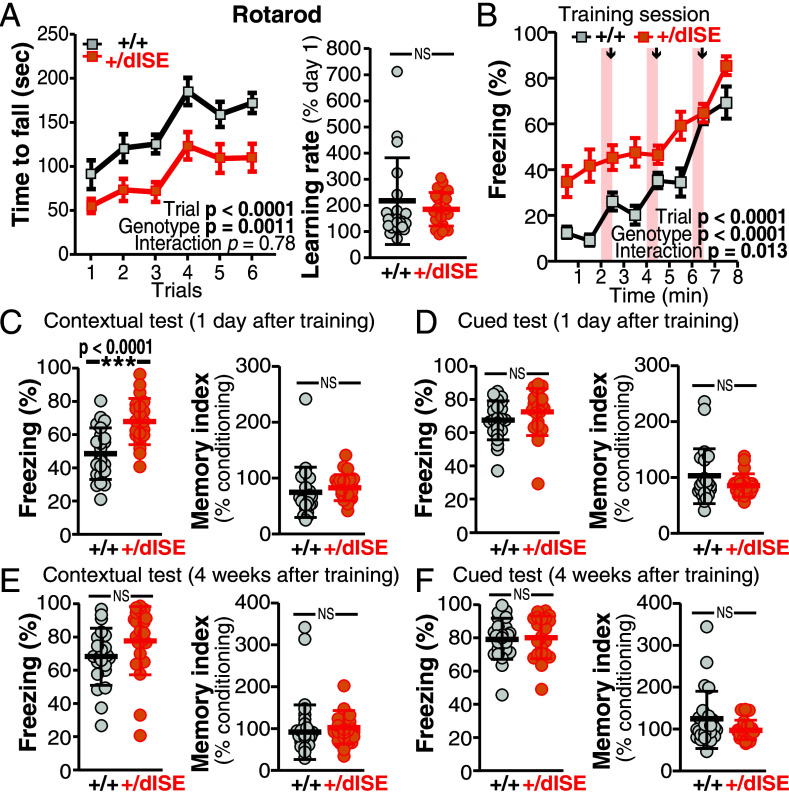
Behavioral abnormality in *Ptprd^+/dISE^* mice. (*A*) Motor coordination and learning of *Ptprd^+/dISE^* mice in rotarod test with six trials. Latency to fall off the rotarod (*Left*) in each trial and learning rates given by dividing the average latency to fall-off in the 1st to 3rd three trials done on Day 1 by that in the 4th to 6th three trials on Day 2 (*Right*). (*B–F*) Contextual and cued fear conditioning and memory of *Ptprd^+/dISE^* mice. Freezing responses during conditioning with footshocks of 0.3 mA (*B*), in contextual (*C* and *E*) and cued (*D* and *F*) tests conducted 1 d (C and *D*) and 4 wk (*E* and *F*) after conditioning. Memory index, which is given by dividing the freezing rate in the test session by that in the last conditioning session, is shown *Right* in each panel. Data are mean ± SEM. for (*A*) *Left* and (*B*), and mean ± SD. for (*A*) *Right*, and (*C–F*). ***P < 0.001, Two-tailed *t* test. N = 21 and 23 for *Ptprd^+/dISE^* mice and their WT littermates, respectively.

To address the physiological significance of the AS-mediated regulation of meB, we subjected the heterozygous dISE mutant (*Ptprd^+/dISE^*) mice to a behavioral test battery covering a broad range of various behavioral domains, such as sensory-motor functions, emotion, motivation, and learning and memory (24). As a control for behavioral testing, we used heterozygous *Ptprd* knockout (KO) mice (*Ptprd^+/^^−^*), which showed no apparent developmental delay with an unaltered meB selection rate but their total PTPRD protein level was decreased by ~50% compared to that of WT littermates (*SI Appendix*, Fig. S7). Table 1 summarizes the results of the behavioral tests of heterozygous dISE mutant (*Ptprd^+/dISE^*) and KO (*Ptprd^+/−^*) mice. The *Ptprd^+/dISE^* mutant mice exhibited abnormalities in almost all tests. The increased hot-plate latency and decreased acoustic startle responses in the *Ptprd^+/dISE^* mice suggested hypoesthesia (*SI Appendix*, Fig. S8 *A* and *B*). Furthermore, these mice showed a longer latency to enter the light box in the light/dark transition test, decreased stay time in, and a lower number of entries into the open arms in the elevated plus maze test, and spent less time in the center zone in the open-field test, indicating increased anxiety (*SI Appendix*, Fig. S8 *C–E*). The *Ptprd^+/dISE^* mice also showed decreased locomotor activity in the open-field (*SI Appendix*, Fig. S8*E*) and other tests, increased antidepressive behavior in the forced swim test (*SI Appendix*, Fig. S8*F*), slightly reduced sociability (*SI Appendix*, Fig. S9 *A*, *B*, and *E*), and defective motor coordination in the rotarod test (Fig. 4*A*). In contrast, the heterozygous *Ptprd* KO (*Ptprd^+/–^*) mice with unaltered meB selection rate and ~50% decreased total PTPRD protein level exhibited minimal behavioral abnormalities, including a lower hot-plate latency, slight increase in anxiety, and changes in sociability (*SI Appendix*, Figs. S8 *A* and *C* and S9 *D* and *E*). These results suggest that meB selection rates (i.e., the proportion of *Ptprd* splice variants) rather than PTPRD protein levels are crucial for behavioral regulation.

**Table 1.** Results of behavioral test battery of *Ptprd^+/dISE^* and *Ptprd^+/–^* mice

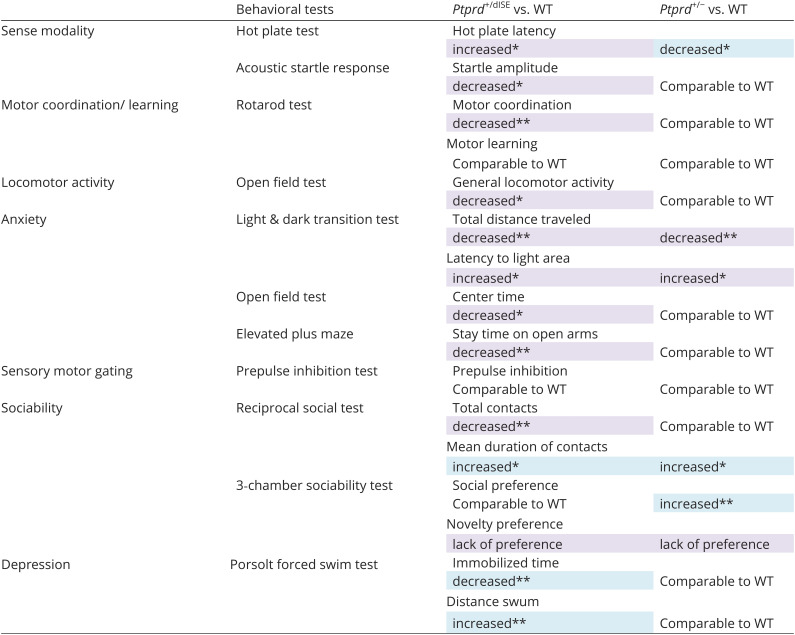

^*^Statistical significance of *P* < 0.05 in two-tailed *t* test.

^**^Statistical significance of *P* < 0.01 in two-tailed *t* test.

Despite severe sensory, motor, motivation, and emotional defects in *Ptprd^+/dISE^* mice, no apparent abnormalities were observed in the learning and memory tasks. In the rotarod test, three trials per day are conducted over 2 d, and the performance normally improves significantly between the first and second day (25). Even though the *Ptprd^+/dISE^* mice exhibited impaired motor coordination, as indicated by decreased retention time on the rotarod throughout the trials, their performance obviously improved between the first and second day. Furthermore, the learning rates of these mutant mice were comparable to those of their WT littermates (Fig. 4*A*). Next, we conducted a fear conditioning task to address contextual and cued fear learning and memory. The *Ptprd^+/dISE^* mice showed increased freezing level during the conditioning session even before the 1st footshock, suggesting reduced locomotor activity of these mutants in novel environments, as seen in all other behavioral tests (Fig. 4*B*). In the contextual and cued tests conducted 1 d and 4 wk after conditioning, the *Ptprd^+/dISE^* mice showed higher or comparable freezing levels vs. their WT littermates (Fig. 4 *C–F*). The ability of the mice to retain fear memory was evaluated using a memory index, which is a normalized ratio based on % freezing during the last 1 min of the conditioning session. The memory indices were comparable between the *Ptprd^+/dISE^* and WT mice in all the contextual and cued tests, suggesting that both recent and remote fear memory were well established and retained in *Ptprd^+/dISE^* mice (Fig. 4 *C–F*). Furthermore, the *Ptprd^+/dISE^* mice showed unaltered spatial learning and memory in the Barnes maze test (*SI Appendix*, Fig. S10).

### Identification of ISS Region for meB.

**Fig. 5.**
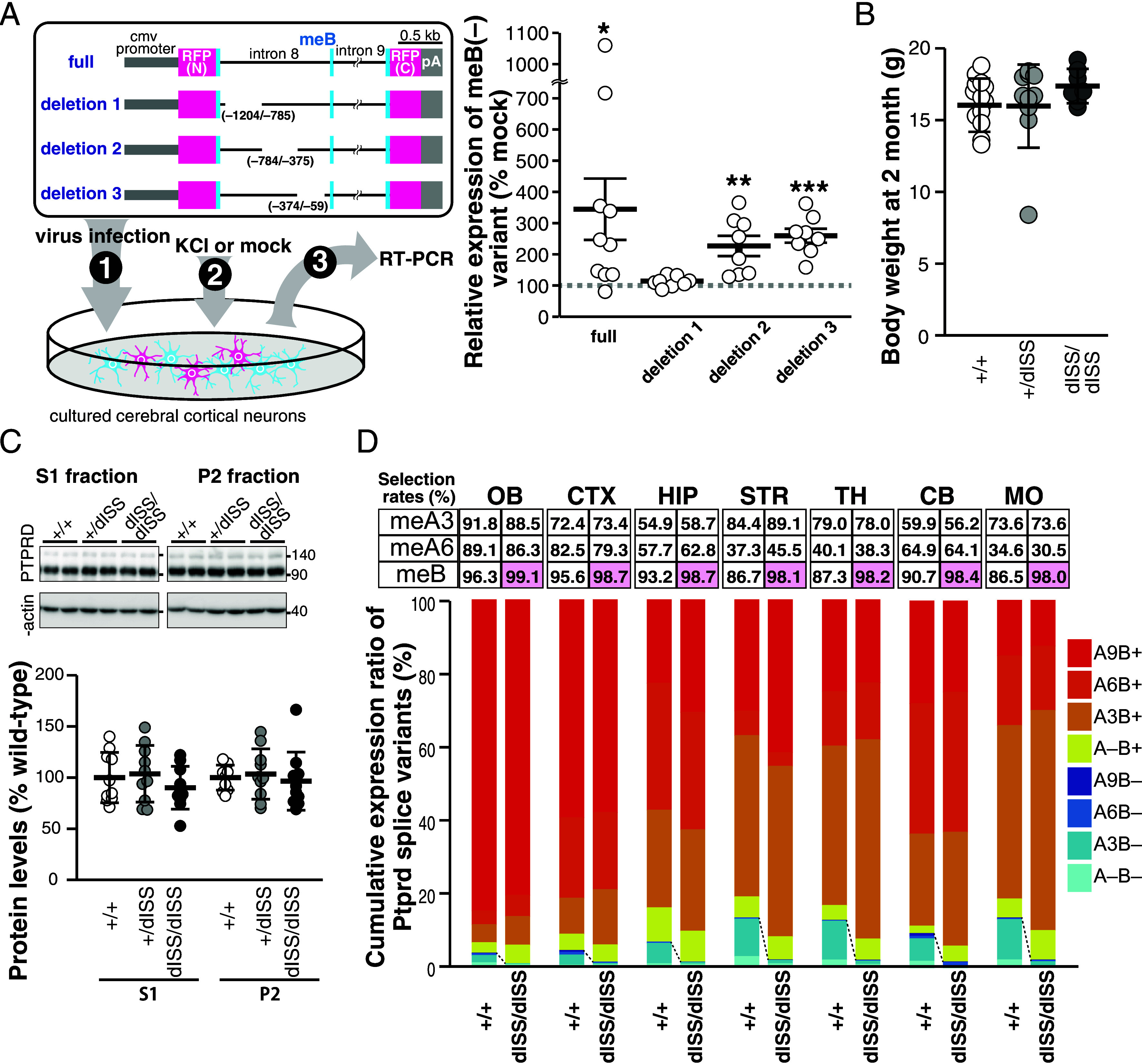
Identification of ISS region for *Ptprd* meB. (*A*) Schematic presentation of reporter minigenes and screening system for neuronal activity–dependent ISS region for meB (*Left*) and quantification of activity-dependent meB skipping of each reporter minigene (*Right*) (n = 10, 8, 8, and 8 experiments for full, deletion 1, deletion 2, and deletion 3 minigenes, respectively). (*B*) Body weights of 8-wk-old homozygous (N = 7) and heterozygous (N = 12) *Ptprd^dISS^* mutant mice and their WT littermate (N = 13). (*C*) Representative immunoblots (*Top*) and quantification of expression levels (*Bottom*) of total PTPRD protein in the whole brain S1 fraction and synaptosomal P2 fraction in *Ptprd^+/dISS^, Ptprd^dISS/dISS^*, and their littermate WT mice (N = 10 each). (*D*) Selection rates of meA3, meA6, and meB (*Top*) and relative abundance ratios of eight *Ptprd* splice variants (*Bottom*) in the OB, CTX, HIP, STR, TH, CB, and MO of 8-wk-old WT (*Ptprd*^+/+^), heterozygous dISS mutant, and homozygous dISS mutant mice (N = 4, each). Statistically significant differences in two-tailed *t* test are shaded in pink. Data are mean ± SD for (*B* and *C*), and mean ± SEM for (*A*).

The robust learning ability and memory of the *Ptprd^+/dISE^* mutant mice suggested that intronic region(s) besides the 316-bp ISE region might regulate these functions by modulating neuronal activity–dependent meB skipping. In fact, KCl (i.e., neuronal activity)-induced skipping of meB was observed in cultured neurons prepared from the *Ptprd^+/dISE^* and *Ptprd^dISE/dISE^* mice (*SI Appendix*, Fig. S11). Therefore, we searched for element(s) that facilitate neuronal activity–dependent meB skipping by introducing meB splicing reporter minigenes with various intronic deletions into the cultured neurons (Fig. 5*A*). These neurons were treated with KCl and subjected to real-time quantitative PCR to detect the transcripts lacking meB from the minigenes. The results revealed that deletion of a 420-bp sequence located –1,204 to –785 bp upstream of meB (deletion 1) completely suppressed KCl-induced skipping of meB. Further dissection of this 420-bp sequence revealed that a putative hnRNPL binding motif contributes to this regulation (*SI Appendix*, Fig. S12 *A* and *B*). We further found that pharmacological inhibition of calcium/calmodulin-dependent protein kinase II/IV by KN93 suppressed KCl-induced skipping of meB (*SI Appendix*, Fig. S12*C*).

To further validate that the 420-bp sequence functions as splicing silencer for meB in vivo, we generated *Ptprd* mutant mice lacking this sequence (*Ptprd^dISS^*) via CRISPR/Cas9-mediated genome editing. The heterozygous (*Ptprd^+/dISS^*) and homozygous (*Ptprd^dISS/dISS^*) deletion of this sequence did not affect the growth of the mice (Fig. 5*B*) and total PTPRD protein expression levels in the brain (Fig. 5*C*). Analysis of the distribution patterns of the eight *Ptprd* splice variants in the brain of WT and *Ptprd^dISS/dISS^* mice confirmed that the meB selection rates were increased to nearly 100% in all brain regions examined (Fig. 5*D*). These results strongly suggest that the 420-bp intronic region functions as an ISS for meB. To examine the consequence of the ISS deletion on developmental and activity-dependent synaptogenic properties, we utilized the primary neuronal coculture system from WT and *Ptprd^dISS/dISS^* mice. Compared to WT neurons showing increments of meB peptide-lacking PTPRD variants/ NLGN3-mediated synaptogenesis after KCl treatment, *Ptprd^dISS/dISS^* neurons failed to show synaptogenic activity against NLGN3 even after KCl treatment (*SI Appendix*, Fig. S13). In contrast, meB containing PTPRD variants/IL1RAPL1-mediated synaptogenesis was comparable between WT and *Ptprd^dISS/dISS^* neurons.

### Selective Impairments in Learning and Memory by ISS Disruption.

**Fig. 6.**
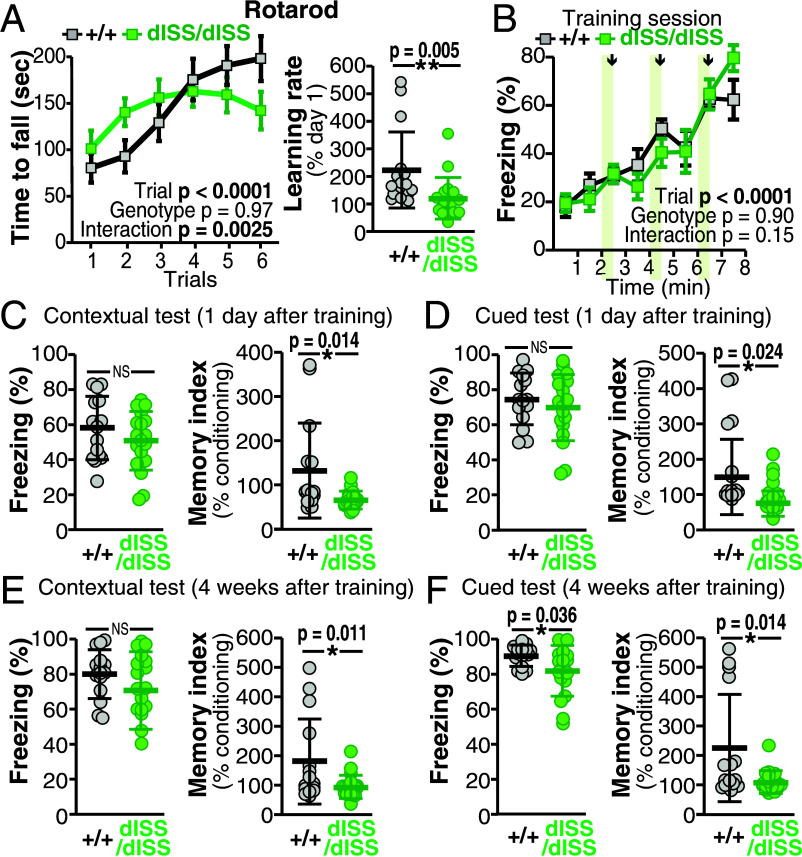
Selective impairment in learning and memory in *Ptprd^dISS/dISS^* mice. (*A*) Motor coordination and learning of *Ptprd^dISS/dISS^* mice in rotarod test with six trials. Latency to fall off the rotarod (*Left*) in each trial and learning rates given by dividing the average latency to fall-off in the 1st to 3rd three trials done on Day 1 by that in the 4th to 6th three trials on Day 2 (*Right*). (*B–F*) Contextual and cued fear conditioning and memory of *Ptprd^dISS/dISS^* mice. Freezing responses during conditioning with footshocks of 0.3 mA (*B*), in contextual (*C* and *E*) and cued (*D* and *F*) tests conducted 1 d (*C* and *D*) and 4 wk (*E* and *F*) after conditioning. Memory index is shown right in each panel. Data are mean ± SEM. for (*A*) *Left* and (*B*), and mean ± SD for (*A*) *Right*, (*C–F*). *P < 0.05 and **P < 0.01, Two-tailed *t* test. N = 18, and 16 for *Ptprd^dISS/dISS^* mice and their WT littermates, respectively.

To further address the physiological significance of the ISS for meB, we used the behavioral test battery for the homozygous mutants (*Ptprd^dISS/dISS^*) and their WT littermates. The performance of the *Ptprd^dISS/dISS^* mice was comparable to that of their WT littermates in almost all behavioral tests (*SI Appendix*, Fig. S14 and Table S1). However, the mutant mice had difficulties in learning and memory. In the rotarod test, the *Ptprd^dISS/dISS^* mice showed rather higher performance on the rotating rod on Day 1, however, no improvement in performance was observed between Days 1 and 2, suggesting impaired motor learning (Fig. 6*A*). This was in good contrast with the *Ptprd^+/dIES^* mice, which exhibited a robust motor learning ability, albeit with impaired motor coordination (Fig. 4*A*).

In the Barnes maze test, no significant differences were observed between the genotypes during training session and probe tests, suggesting that spatial reference learning and memory were unaltered in the *Ptprd^dISS/dISS^* mice (*SI Appendix*, Fig. S14*I*).

In the contextual and cued fear conditioning test, the freezing level of *Ptprd^dISS/dISS^* mice increased as efficiently as did that of their WT littermates during the conditioning session, suggesting no obvious changes in the fear learning process (Fig. 6*B*). The freezing levels were comparable between genotypes in the contextual tests conducted 1 d and 4 wk after conditioning and in the cued test conducted 1 d after conditioning. However, these levels were significantly lower in the *Ptprd^dISS/dISS^* mice in the cued test 4 wk after conditioning, suggesting impaired remote cued fear memory formation (Fig. 6 *C–F*). Since memory indices, which are ratios of freezing during conditioning and during the test, were lower in *Ptprd^dISS/dISS^* mice in all of the contextual and cued tests, these mice may have difficulties in fear memory retention (Fig. 6 *C–F*). These results suggest that activity-dependent meB skipping is a key process for the facilitation of specific types of learning and memory.

## Discussion

Microexons are mostly neuronal-specific and highly conserved among vertebrates. The AS events of these microexons contribute to transcriptomic and proteomic diversity and complexity, and their dysregulation has been implicated in neurodevelopmental disorders (6, 7, 26–28). Three microexon (meA3, meA6, and meB)-derived peptides of PTPRD, which is a hub presynaptic organizer, play a crucial role in the regulation of synaptogenic activity and synaptic property via their insertion into interfaces with postsynaptic binding partners. Thus, the AS programs for these three microexons provide each of the eight resulting splice variants with distinct synaptogenic property (16). In this study, we demonstrated that a tiny 12-nt microexon, meB of *Ptprd*, is spatiotemporally and neuronal activity–dependently regulated by AS, and that the AS code is crucial for shaping mouse behaviors.

Choices of *Ptprd*-meB demonstrated different contributions to neuronal cultures from different brain regions, as meB peptide determined synaptic properties, i.e., excitatory or inhibitory, in cerebral cortical and hippocampal neurons while the same peptide determined synaptogenic activity in cerebellar neurons (Fig. 1 *C* and *D* and *SI Appendix*, Fig. S1). The eight *Ptprd* splice variants generated by alterative choices of meA3, meA6, and meB showed distinct brain region-specific distribution patterns with minor interindividual variations (Fig. 2 *A* and *C*) in vivo, suggesting that the abundance ratio of these variants is genetically regulated and essential for brain function. The abundance ratios of the eight splice variants further changed developmentally in the CTX and CB (Fig. 2 *B*, *D*, and *E*), indicating the brain region specific contribution of AS programs for *Ptprd* microexons to the assembly and remodeling of the neural circuits that is necessary for brain maturation. Interestingly, the proportion of meB-lacking *Ptprd* variants decreased in the CB of the mice with age, but not in the CTX, suggesting that the *Ptprd* meB is differently involved in neuronal maturation and its process might differ in these brain regions.

The meB-containing *Ptprd* variants were predominantly expressed in all brain regions studied, especially in the OB and CTX. These results are in line with recent report by Han et al. (20). using RNA-Seq and quantitative real-time PCR techniques to analyze meA and meB insertion separately. Because each PTPRD splice variant has a distinct synaptogenic property (16), it is presumable that the region specific abundance ratio of the eight splice variants contributes to the formation of a specific neural circuit for a particular function. Furthermore, in the cultured cerebral cortical neurons, the relative ratios of the eight *Ptprd* splice variants were altered by KCl treatment, i.e., by neuronal activity (Fig. 2 *F–I*), which seems to have been partly caused by the neuronal activity–dependent skipping of meA3 and meB (Fig. 2*F*). Together with our finding that the *Ptprd* microexons determine the synaptogenic properties of PTPRD (Fig. 1 *C* and *D* and *SI Appendix*, Fig. S1) (16) these results support the idea that the AS-mediated regulation of *Ptprd* microexons functions as genetic and environment-dependent blueprint for synapse formation.

The deletion of the 316-bp intronic sequence encompassing two consensus intronic splicing enhancer UCUCU/UGC motifs (23) in *Ptprd^dISE^* mice caused a dose-dependent decrease in meB (exon 9)-containing variants in all brain regions examined. However, the decreasing pattern of the *Ptprd* meB-containing transcripts was not uniform, and the switching pattern from *Ptprd* meA(9, 6, 3, –)/B(+) to *Ptprd* meA(9, 6, 3, –)/B(–) varied among different brain regions. This indicates that individual neurons have specific *Ptprd* microexon selection patterns, and the contribution of the 316-bp intronic sequence to meB selection is distinct in each neuron. Indeed, in cultured cortical neurons, the meB proximal UCUCU sequence contributes significantly to meB selection (*SI Appendix*, Fig. S4). However, other putative binding elements for splicing factors and RNA binding proteins such as RBFOX1, PCBP3, HNRNPH2, and HNRNPA2B1, are also present in this 316-bp sequence (http://rbpmap.technion.ac.il), which may explain the different contributions of this 316-bp sequence across brain regions. Because intron 8 is well conserved across species over a stretch of approximately 1.3 kb (e.g., 71.7% sequence identity between mice and humans), including this 316-bp region, neuron and/or brain region-specific splicing regulatory elements may densely reside in this intronic region. In fact, activity-dependent skipping of meB was still observed in the neurons from homozygous *Ptprd^dISE^* mutant mice (*SI Appendix*, Fig. S11). Therefore, we screened the 420-bp intronic sequence near exon 8 as the responsible region for this regulation (Fig. 5*A*). It has been proposed that the activity-dependent skipping of microexons is mediated by a shift to the expression of nonproductive *nSR100* transcripts and proteolytic degradation of nSR100/Srrm4 protein, a major neuronal splicing regulator acting on the consensus ISE elements (23, 29). In contrast, the 420-bp region we found in this study rather functions as a splicing suppressor/silencer with a novel regulatory mode, which induces rapid exclusion of downstream microexon within 1 h of neuronal activation (Fig. 2*H*). Furthermore, it seems that all of the splicing silencer elements for meB are included within this 420-bp region, since the lack of this region almost completely suppressed meB skipping in all brain regions (Fig. 5*D*). The putative hnRNPL binding sequence within this intron region appears to be involved, at least in cortical neurons (*SI Appendix*, Fig. S12), but it is also possible that RBFOX, BRUNOL, CPEB2, DAZ3, HuR, and PTBP3, which are predicted to bind to this region (http://rbpmap.technion.ac.il), are also broadly involved in this regulation.

Misregulation of the AS of microexons has been implicated in neurological diseases, including ASD and schizophrenia (7, 30, 31). Accordingly, decreased expression of the major neuronal microexon splicing regulator nSR100/SRRM4 is observed in autistic brain (7) and causes ASD-like behavioral abnormalities in mice (29). Furthermore, an overexpression-mediated imbalance in the microexon isoform of cytoplasmic polyadenylation element binding protein 4 (CPEB4), which orchestrates the translation of multiple ASD risk genes by modulating their poly(A)-tails, causes ASD-related behavioral phenotype in mice (32). Moreover, it has been demonstrated that a deletion of the translation initiation factor *Eif4g1* microexon, which is activity-dependently regulated and misregulated in autistic brain, causes deficits in social behavior, learning, and memory (33). However, little is known about the comprehensive effects of the dysregulation of the AS of individual microexons on a wide range of behavioral domains. In this study, we utilized a behavioral test battery to systematically evaluate the effects of the dysregulation of the genetic and neuronal activity–dependent programs for the *Ptprd* meB selection. The *Ptprd^+/dISE^* mice heterozygously lacked the 316-bp intronic splicing enhancer region for meB, resulting in approximately 25% decrease on average in meB selection rates across different brain regions with unaltered total PTPRD protein levels. Meanwhile the *Ptprd^dISS/dISS^* mice with a homozygous deletion of the 420-bp intronic region responsible for neuronal activity–dependent meB skipping exhibited almost complete loss of the splice variants lacking meB in the brain with no noticeable change in total PTPRD protein levels. Strikingly, the *Ptprd^+/dISE^* mice demonstrated abnormalities in 10 out of 12 behavioral tests, showing decreased motor coordination and locomotor activity, attenuated sociability, increased fear/anxiety-related behaviors, antidepression-like behaviors, and hypoesthesia (Table 1). In contrast, heterozygous *Ptprd* KO (*Ptprd^+/–^*) mice with approximately 50% reduction in total PTPRD protein and normal abundance ratio of the eight splice variants performed better in the same behavioral test set (Table 1). These results indicate that the appropriate proportions of the eight *Ptprd* splice variants are more important than the absolute amount of PTPRD protein in shaping behavior in mice. Besides, the *Ptprd^+/dISE^* and *Ptprd^+/–^* mice partly shared behavioral characteristics (i.e., increased anxiety in the light/dark transition test and decreased novelty preference in the 3-chamber-sociability test) and the *Ptprd^+/dISE^* mice consistently showed a decreased ambulatory activity in all behavioral test, suggesting that the absolute amount of meB-containing PTPRD variants is also important for the fundamental neuronal function. As mentioned above, *Ptprd^+/dISE^* mice lack the Srsf11/Srrm4 consensus sequence and exhibit an ASD-related endophenotype, an E/I imbalance in the CTX, suggesting that dysregulation of meB may be involved in ASD-like behaviors caused by Srrm4 deficiency (29). Despite the number of behavioral abnormalities in *Ptprd^+/dISE^* mice, they still showed robust ability in motor learning (rotarod test), spatial reference memory (Barnes maze test), and associative fear learning and memory (fear conditioning test), possibly because the neuronal activity–dependent splicing regulatory region was intact. As expected, the *Ptprd^dISS/dISS^* mice were specifically impaired in motor learning and contextual and cued fear memory (Fig. 6 and *SI Appendix*, Table S1), indicating that activity-dependent *Ptprd* meB skipping is part of underlying process of some forms of long-term learning and memory. Memory is formed, consolidated, stored, retrieved, and forgotten. These dynamic processes occur in temporal sequences. Motor skill learning is associated with substantial recruitment of neurons in the M1 area (34) and with synapse formation between the M1 and M2 areas or the TH (35, 36). The number of synapses in the lateral amygdala is reported to be increased in the fear conditioned animals, suggesting the involvement of synaptogenesis in fear acquisition (37, 38). PTPRD variants containing the meB peptide mediate synapse formation through postsynaptic IL1RAPL1, IL-1RAcP, and Slitrks, whereas those lacking the meB peptide do so through NLGN3 (11–13, 16, 39). Therefore, this ligand switching triggered by activity-dependent *Ptprd* meB skipping may be responsible for the learning and memory-associated synapse formation and rearrangement. Indeed, increment of NLGN3-PTPRD mediated synaptogenesis occurs by 24 h after neuronal activation (Fig. 2*I*), which seems consistent with the time course of motor learning and fear memory formation. These pliable changes in synaptogenic property could be the art of microexons’ splicing code. Although the regulation by splicing codes is unknown, Han et al. reported that subicular neuron-selective *Ptprd* meA3 and meA6 deletion led to abnormalities in object-location memory, suggesting selection of *Ptprd* meA should be regulated by splicing code for shaping ideal behavior fitting to the environment. Splicing codes for *Ptprd* meA3, meA6, and meB should collaborate in harmony with each other being affected by environmental stimuli and the orchestration needs to be studied.

In this study, we showed that a single microexon in the *Ptprd* gene, which encodes a peptide of only four amino acids was selected based on both the genetic AS program and the neuronal activity–dependent AS program by environmental triggers to create a relative ratio of PTPRD splice variants, thereby shaping mouse behavior. An intriguing possibility is that these AS programs for *Ptprd* microexons may confer pliability to the expression ratio of PTPRD variants throughout life, forming the basis of individual personality. Unraveling the genetic and activity-dependent AS programs for *Ptprd* microexons at the single cell level in the brain may help elucidate the architectural plan for neural connections formed by individual neurons to shape an elaborate behavioral regulatory system.

## Materials and Methods

### Animal Experiments.

All the procedures were approved by the Animal Experiment Committee of the University of Toyama (Authorization No. A2023MED-05 and A2016OPR-3) and conducted in accordance with the Guidelines for the Care and Use of Laboratory Animals of the University of Toyama. Mice were housed in a room under a 12 h light/dark cycle (lights on at 7:00 a.m.) at 23 ± 3 °C and 30 to 60% humidity. Food and water were provided ad libitum. Both male and female mice were used for biochemical and expression analyses. For behavioral testing, the male offspring of mating pairs were weaned around 1 mo, genotyped, and housed 4 (two pairs of WT and mutant mice) per cage.

### Generation of *Ptprd^dISE^*, *Ptprd^dISS^*, and *Ptprd* KO Mice.

The electroporation and microinjection of guide RNA, Cas9 protein, ssODN, and targeting vector into C57BL/6N mouse zygotes were performed essentially according to the previous reports (40–42) and detailed procedures are described in *SI Appendix*, *Supplementary Methods*.

### Cell Cultures and Coculture Assay.

Preparation of primary hippocampal, cerebral cortical, and cerebellar neurons and subsequent neuron-bead cocultures followed by immunostaining were performed essentially as described previously (11, 16, 43), and detailed procedures are described in *SI Appendix*, *Supplementary Methods*.

### Analyses of *Ptprd* Microexons’ Splicing Patterns (meA/B Profiling).

First-strand cDNA was synthesized from total RNAs extracted from brain tissues and primary cultured neurons, and subjected to *Ptprd* cDNA fingerprinting to analyze the ratios of eight splice variants. Detailed procedures are described in *SI Appendix*, *Supplementary Methods*.

### Splicing Reporter Assay.

The cytomegalovirus promoter and rat synapsin1 promoter-driven tagRFP gene separated by the *Ptprd* genomic DNA fragment covering intron 8 with various mutations, exon 9 (meB), and intron 9 was constructed for packaging of adeno associated viruses (AAVs). cDNAs from the AAV-infected primary cortical neurons were subjected to a quantitative real-time PCR for meB-lacking transcript or to cloning of the tagRFP gene fragment to quantify the ratio of meB-containing and meB-lacking transcripts. The detailed procedures are described in *SI Appendix*, *Supplementary Methods*.

### Western Blotting.

The S1 and P2 fractions were prepared from 6-wk-old male and female mice as previously described (44), and protein concentrations were quantified using the BCA protein assay kit (PIERCE, Rockford, United States). Equal amounts of protein were separated by SDS-PAGE, transferred to PVDF membranes and probed with mouse anti-PTPRD [Abcam (ab233806), 1:500 dilution] antibody followed by horseradish-peroxidase-conjugated secondary antibody. The blots were developed and imaged using a Luminescent Image Analyzer LAS-4000 mini (Fujifilm, Tokyo, Japan). Blots were stripped and reprobed with mouse anti-β-actin [Santa Cruz Biotechnology (sc-47778), 1:1,000 dilution] antibody to normalize for differences in loading. The relative levels of each protein were determined by densitometric analysis using serially diluted protein samples on the same blots. For *Ptprd^dISE^, PtprdKO*, and *Ptprd^dISS^*, three independent experiments using more than three litters in total were performed and pooled for densitometric analysis. Densitometric analysis of western blots was performed using Image J 1.46 software (45).

### Behavioral Test Battery.

All the behavioral experiments were carried out with male mice essentially according to the previous report (19) and detailed procedures are described in *SI Appendix*, *Supplementary Methods*.

#### Rotarod test.

Rotarod test was conducted using an accelerating rotarod apparatus (UGO Basile, Comerio-Varese, Italy) according to a previous report (25). Mice were placed on a rotating drum and the latency to fall-off was recorded with 5 min cutoff. Rotarod testing consisted of six trials with three trials per day over the course of 2 d. The speed of the drum accelerated from 4 to 40 rpm over a 5 min period for a trial.

#### Fear conditioning.

Contextual and cued fear conditioning using 0.3 mA footshock were conducted with mice used for behavioral test battery as described previously (46). The tests consisted of three sessions, conditioning (Day 1), and contextual and cued tests (Day 2 and 30). In the conditioning session, a 55 dB white noise, which served as the conditioned stimulus (CS), was played for 30 s from 120, 240, and 360 s. During the last 2 s of the tone, a footshock of 0.3 mA was delivered as the unconditioned stimulus (US). Each mouse received three CS-US pairings with 2 min interstimulus interval. Contextual testing was conducted 24 h after conditioning (Day 2). The mice were placed in the same chamber that contextual conditioning had taken place and monitored for freezing for 5 min. Cued testing with altered context was conducted 3 h after contextual test in a triangular box (35 × 35 × 40 cm) made of white opaque Plexiglas with illumination level 30 lx. Freezing behavior was assessed during a 3 min free exploration, followed by a 3 min presentation of the tone. Contextual and cued tests were conducted again at Day 30 to assess remote memory. Percentage of freezing time was measured using ImageFZ software with image capture rate of 2 frame/s.

### Quantification and Statistical Analysis.

All numerical data are processed statistically. Datasets of meA/B profiling were analyzed using R statistical software (version 4.4.0). Data were analyzed by the Dunnett test, PCoA with 95% CI. Behavioral data were analyzed using StatView (ASA Institute, Cary, NC, United States). Comparison between control and mutant mice were conducted by one-way ANOVA followed by Tukey’s test, two-way repeated measures ANOVA followed by Fisher’s LDS test, Dunnett’s test, Student’s *t* test, paired *t* test, or Mann–Whitney’s *U* test. Statistical significance was assumed when *P* < 0.05.

## Supplementary Material

Appendix 01 (PDF)

Dataset S01 (XLSX)

Dataset S02 (XLSX)

## Data Availability

Study data are included in the article and/or supporting information.
