# Supplementary material for: Alternative microexon splicing code for a four-amino acid peptide of PTPRD governs behavioral development
Source: Proc Natl Acad Sci U S A. 2026 Apr 8;123(15):e2515310123. doi: 10.1073/pnas.2515310123 (PMC13080001; doi:10.1073/pnas.2515310123)
Supplement: Supplementary file 1 — Appendix 01 (PDF) [file pnas.2515310123.sapp.pdf]

Supporting Information for

Alternative microexon splicing code for a four-amino-acid peptide of PTPRD governs behavioral development.

Ayako Imai[1,2,3], Hironori Izumi[1,2], Nagomi Ito[4], Hina Ogiso[5], Yuki Kitajima[6], Shuhei Kawase[6], Mizuki Sendo[1], Kenji Azechi[1], Toshihide Tabata[6], Yumie Koshidaka[7], Shuya Fukai[8], Keizo Takao[2,7], Hisashi Mori[1,2] & Tomoyuki Yoshida[1,2*].

[1] Department of Molecular Neuroscience, Graduate School of Medicine and Pharmaceutical Sciences, University of Toyama, Toyama 930-0194, Japan
[2] Research Center for Idling Brain Science, University of Toyama, Toyama 930-0194, Japan
[3] Department of Veterinary Nursing and Health Science, School of Veterinary Medicine, Azabu University, Kanagawa 252-5201, Japan
[4] Interdisciplinary Graduate School of Medicine, Pharmacy, Sciences and Engineering, University of Toyama, Toyama 930-0194, Japan
[5] Faculty of Engineering, University of Toyama, Toyama 930-8555, Japan
[6] Laboratory for Biological Information Processing, Graduate School of Science and Engineering, University of Toyama, Toyama 930-8555, Japan
[7] Life Science Research Center, University of Toyama, Toyama 930-0194, Japan
[8] Department of Chemistry, Graduate School of Science, Kyoto University, Kyoto 606-8502, Japan

* **Corresponding author:** Tomoyuki Yoshida
Email: toyoshid@med.u-toyama.ac.jp

This PDF file includes:

Supporting text
Figures S1 to S14
Tables S1
Legends to Figures S1 to S14
SI References

Supporting Information Text

Supplemental methods

Generation of *Ptprd*^{dISE} and *Ptprd*^{dISS} mice

The electroporation of guide RNA, Cas9 protein, and ssODN into C57BL/6N mouse zygotes was performed as previously described (1, 2). The sequences of guide RNAs (gRNA-meB-ISE-5' and gRNA-meB-ISE-3') for generation of *Ptprd*^{dISE} line were 5'-CAACUCCACAGAGAGAAAGG-3' and 5'-GAGGAAAAGGAGAAAAACAA-3'. The sequences of guide RNAs (gRNA-meB-ISS-5' and gRNA-meB-ISS-3') and ssODN (meB-dISS-ssODN) for generation of *Ptprd*^{dISS} line were 5'-GGGGAGUUGAGUUGCUCACC-3', 5'-GGAAGCUAAGCGUAAGAGAA-3' and, 5'-CAGAGGTAGGAATGACATCACCCTGGCATGGCGTCTCCATTCAGGCTCAGGGGGACTTTCT CTTACGCTTAGCTTCCTAATTAAAACCCAACTTTATATAAGTAAATGCA-3', respectively. The genomic DNAs from the fingers of F0 mice were subjected to PCR using primer sets, 5'-TTTCTCTTACGCTTAGCTTCC-3' and 5'-TCATGCATTGCATTTGGACG-3', and 5'-ACTAGTTTATATGTCAGAGGTAGGAATGAC-3' and 5'-AAAGGAGAAAAAAGGGGGAAAAACAGCATCC-3', to detect deletions of the ISE and ISS sequences, respectively. The PCR fragments were sequenced to confirm the ssODN-mediated precise deletion events. The F0 mosaic mice were crossed with WT C57BL6N mice to generate heterozygous (*Ptprd*$^{+/dISE}$ and *Ptprd*$^{+/dISS}$) F1 mice. These F1 mice were backcrossed twice with WT C57BL/6N mice, and subsequent generations were maintained by mating between heterozygous mice.

Generation of *Ptprd* knockout mice

The 1647 bp *Ptprd* intronic sequence, 108 bp mouse interleukin-2 receptor gene, 720 bp venus gene, 209 bp SV40 polyadenylation signal sequence, and 251 bp *Ptprd* intronic sequence were amplified with PCR with primers, 5'-TAAGCTTAGCCAGAGTTCCCAGACAGAATTCC-3' and 5'-CTCCATACGGCGAACTGGAACAAAACACAAGG-3', 5'-TTCCAGTTCGCCGTATGGAGTATAAGGTAG-3' and 5'-GCTCACCATGATGGTTCTTCTGCTCTTCC-3', 5'-

GAAGAACCATCATGGTGAGCAAGGGCGAGG-3’ and 5’-GAGTGCGGCCGCTTACTTGTACAGCTCGTCCATG-3’, 5’-CAAGTAAGCGGCCGCACTCTAGATCATAATCAGCC-3’ and 5’-GAGAATCCTCGAGAAACCACAACTAGAAT-3’, and 5’-GTGGTTTCTCGAGGATTCTCTATCCCACCCAC-3’ and 5’-CATACCTTTGACAGTGATCTGGG-3’, respectively, connected by PCR with primers 5’-TAAGCTTAGCCAGAGTTCCCAGACAGAATTCC-3’ and 5’-CATACCTTTGACAGTGATCTGGG-3’, and cloned into pBluescript II vector (Stratagene) to yield a targeting vector. The targeting vector, guide RNAs (5’-GUGGGAUAGAGAAUCUUGGU-3’, 5’-CAUACCUUUGACAGUGAUCU-3’, and 5’-AUUCUAGUUGUGGUUUCUCG-3’) and Cas9 protein were injected into C57BL/6N mouse zygotes essentially according to a previously described method (3). The genomic DNAs from the fingers of F0 mice were amplified by PCR with primers, 5’-TTCACTGCATTCTAGTTGTGG-3’ and 5’-GACAGAACTAAGAACAGATCC-3’ and confirmed the precise homologous recombination by DNA sequencing. The F0 mosaic mice were crossed with WT C57BL/6N mice to generate heterozygous F1 mice and the strain was maintained by crossing with WT C57BL/6N mice.

Cell cultures and coculture assay

Primary hippocampal, cerebral cortical, and cerebellar cultures were prepared from mice at embryonic day (E) 17, E18, and postnatal day 6, respectively essentially as described previously (4, 5) Neurons were placed on coverslips coated with 30 µg/mL poly-L-lysine and 10 µg/mL mouse laminin at densities of 3×10^5 cells, 3×10^5 cells, and 7×10^5 cells, per well in a 24-well-dish for hippocampal, cerebral cortical, and cerebellar cultures, respectively. The cells were cultured in Neurobasal-A supplemented with 2% B-27 supplement (Invitrogen), 5% fetal calf serum (FCS), 100 U/mL penicillin, 100 µg/mL streptomycin and 0.5 mM L-glutamine for 24 h, and then cultured in the same medium without FCS. Recombinant ECDs of PTPRD variants, NRXN1β, IL1RAPL1, and NLGN3^{HSE} fused with human Fc (6) were incubated with Protein A-conjugated magnetic beads (smooth surface, 4.0–4.5 µm diameter; Spherotech). Beads coupled with Fc or Fc fusion proteins were added to neurons at DIV 2, 7, 13, or 15 to induce pre- or postsynaptic differentiation. After

24 h, cocultures were fixed and immunostained. Rabbit anti-Shank2 (Frontier Institute, Shank2-Rb-Af750, 1:200), and mouse anti-gephyrin (Synaptic Systems, 47011, 1:1000) antibodies were used to evaluate postsynaptic differentiation. Mouse anti-Bassoon (Enzo, SAP7F407, 1:300), rabbit anti-VGluT1 (Frontier Institute, VGluT1-Rb-Af500, 1:200), and goat anti-VGAT (Frontier Institute, VGAT-Go-Af620, 1:200) antibodies were used to evaluate presynaptic differentiation. For KCl treatment and subsequent coculture experiments, cultured cerebral cortical neurons at DIV 7 were treated with 50 mM KCl for 4 h, then cocultured with beads coated with Fc or Fc fusion proteins for 24 h. For pharmacological experiments, cerebral cortical neurons at DIV 7 were treated with 30 μM KN93 for 1 h, followed by 30 μM KN93 and 50 mM KCl for 4 h, and harvested for meA/B profiling as described below. Three or four independent neuronal cultures for each experimental condition were used and quantification of fluorescent signals of cocultures was conducted in a blind manner with respect to recombinant proteins conjugated with beads and experimental conditions (KCl or mock). All the recombinant Fc fusion proteins were prepared as previously described (6).

Analyses of *Ptprd* microexons' splicing patterns (meA/B profiling)

Total RNA was extracted from the olfactory bulb, cerebral cortex, striatum, hippocampus, thalamus, cerebellum, and medulla oblongata of 8-week-old male and female mice using Trizol reagent (Invitrogen, Carlsbad, CA, USA), according to the manufacturer's instruction. Regarding cerebral cortex and cerebellum, we additionally extracted total RNA at postnatal day (P) 0, P7, P14, P28, and P56. First-strand cDNA was then synthesized using SuperScript IV reverse transcriptase (Invitrogen, Carlsbad, CA, USA). *Ptprd* cDNA encoding the entire extracellular domain was amplified by PCR with primers, 5'-TGTGCAGCCAGCGGTAATCCG-3' and 5'-ATCTCCCAAGACAGCAGCACTG-3' and cloned into pBluescript II vector (Stratagene). One hundred and ninety-two clones randomly picked up for each sample were subjected to PCR with primers, 5'-GATTTCTTACCTGTTGACACAAGC-3' and 5'-CATGATTTCATGATTAGTGGGTGG-3', digested with PstI, and electrophoresed to distinguish eight *Ptprd* variants containing or lacking meA3, meA6 and meB. For cDNA fingerprinting in cultured neurons, primary cerebral cortical neurons were prepared from each litter of mice. On DIV 7, the neurons were treated with 50 mM

KCl or mock (addition of water equivalent to 2.5% of the culture volume) for 4 hours, after which cDNA was synthesized.

Real-time PCR

Quantitative real-time PCR was performed using cDNAs prepared from cultured cortical neurons at DIV 7 treated with 50 mM KCl using primer sets, Fos-S (5'-GAGCTGACAGATACACTCCAAGCG-3')/Fos-A (5'-CAGTCTGCTGCATAGAAGGAACCG-3'), Bdnf-S (5'-TGGCTGACACTTTTGAGCAC-3')/Bdnf-A (5'-CAAAGGCACTTGACTGCTGA-3'), and Gapdh-S(5'-CATGGCCTCCAAGGAGTAAGAAAC-3')/Gapdh-A(5'-ATTGTGAGGGAGATGCTCAGTG-3') for *Fos*, *Bdnf*, and *Gapdh*, respectively. All the quantitative real-time PCR reaction was carried out on MX3000P (Stratagene) with One Step TB Green® PrimeScript™ RT-PCR Kit (Takara) to quantify the relative expression levels using the comparative threshold cycle (Ct) method. Ct values of *Fos* and *Bdnf* were normalized by those of *Gapdh*.

Immunocytochemistry for Tau and Fos

Primary cerebral cortical neurons at DIV 7 were treated with 50 mM KCl, fixed immediately or 4 hours later, and then immunostained with rabbit anti-cFos (Millipore ABE457, 1/1000 dilution) and mouse anti-Tau (Chemicon MAB3420, 1/1000) antibodies followed by Alexa Fluor 488 conjugated donkey anti-mouse IgG (Molecular Probes, 1/500) and Alexa Fluor 555 conjugated donkey anti-rabbit IgG (Molecular Probes, 1/500) antibodies for confocal microscopy.

Splicing reporter assay

The cytomegalovirus (CMV) promoter and 5' half of the tagRFP gene, 3' half of the tagRFP gene, and SV40 polyadenylation signal sequence was amplified by PCR with primers, 5'-GCGGCCGCTATTAATAGTAATCAATTACGGGG-3' and 5'-GAGTCCGGAGCTACCACTAGTGGATGGGAAGTTCACCC-3', 5'-GTGGTAGCTCCGGACTCGAGGGCCCTGTGATGCAGAAG-3' and 5'-

GATCAGTTAGTTAACCGGAATTAAGTTTGTGCCCCAG-3’, and 5’-TTCCGGTTAACTAACTGATCATAATCAGCCATAC-3’ and 5’-GCGGCCGCAAACCACAACTAGAATGCAG-3’, respectively, using pTagRFP-C vector (Evrogen, Moscow, Russia) as templates, connected by PCR with primers, 5’-GCGGCCGCTATTAATAGTAATCAATTACGGGG-3’ and 5’-GCGGCCGCAAACCACAACTAGAATGCAG-3’, digested with NotI, and cloned into NotI site of pAAV-CMV vector (TAKARA Bio, Otsu, Japan) to yield pAAV-tagRFP-mcs. The 3.5kb *Ptprd* genomic DNA fragment covering intron 8, exon 9 (meB), and intron 9 was amplified by PCR with primers, 5’-ACTAGTTTATATGTCAGAGGTAGGAATGAC-3’ and 5’-CTCGAGTGGGACACGGCGAACTGGAAC-3’, using mouse genomic DNA as a template, digested with SpeI and XhoI, and cloned into the same sites of the pAAV-tagRFP-mcs vector to yield pAAV-meB reporter. The 420 bp (–1204 to –785 from meB), 410 bp (–784 to –375 from meB), and 316 bp (–374 to –59 from meB) sequences within the intron 8 of the pAAV-meB reporter were deleted by PCR-mediated mutagenesis to yield pAAV-meB-del1, pAAV-meB-del2, and pAAV-meB-del3, respectively. For analysis of meB(+)/meB(–) ratios for Figs. S4 and S11, we used rat synapsin promoter. The CMV promoter of pAAV-meB reporter was replaced with rat synapsin I promoter to yield pAAV-Psyn-meB reporter. The 420 bp (–1204 to –785 from meB) sequence, 316 bp (–374 to –59 from meB) sequence, putative Srsf11/Srrm4, hnRNPL, and RBFOX binding sequences within the intron 8 (see Fig. S4 and S11) of pAAV-Psyn-meB reporter were deleted by PCR-mediated mutagenesis. Adeno associated viruses (AAVs) were packaged using manufacture’s instruction (TAKARA Bio, Otsu, Japan). Mouse cerebral cortical neurons were infected with these AAVs at days in vitro (DIV) 0 and treated with 50 mM KCl for 4 hours at DIV 7. Total RNA was extracted using Trizol reagent (Invitrogen, Carlsbad, CA, USA) and first-strand cDNA was synthesized using SuperScript IV reverse transcriptase (Invitrogen, Carlsbad, CA, USA) for RT-PCR and meB(+)/meB(–) ratio analysis. Quantitative real-time PCR was performed with primer sets 5’-TTGGTGGGACACGGCGAACTC-3’ and 5’-GCTGCCTCATCTACAACGTC-3’ for tagRFP lacking meB (*tagRFP-meB(–)*) and 5’-GTGGTAGCTCCGGACTCGAGGGCCCTGTGATGCAGAAG-3’ and 5’-GATCAGTTAGTTAACCGGAATTAAGTTTGTGCCCCAG-3’ for total tagRFP transcripts

(*pan-tagRFP*). All the quantitative real-time PCR reaction was carried out on AriaMx (Agilent technology, Santa Clara, CA, USA) with GeneAce SYBR qPCR kit (Nippon Gene, Toyama, Japan) to quantify the relative expression levels using the comparative threshold cycle (Ct) method. Ct values of *tagRFP-meB(–)* were normalized by those of *pan-tagRFP*. For meB(+)/meB(–) ratio analysis, tagRFP fragments were amplified by PCR with a primer set 5'-GCTGCCTCATCTACAACGTC-3' and 5'- GCAAGTAAAACCTCTACAAATGTGG-3' and cloned into pTAC-1 vector (BioDynamics) . The resulting 48 colonies of E. coli were analyzed for inclusion and exclusion of meB.

Behavioral test battery

For the $Ptprd^{dISE}$, $Ptprd^{dISS}$ and *Ptprd* knockout lines, heterozygous mice obtained after 3, 3, and 5 backcrosses with the WT C57BL6N mice, respectively, were used as founders to obtain mice for behavioral experiments. The behavioral test battery included general health and neurological screening, light/dark transition test, open field test, elevated plus maze test, rotarod test, hot plate test, reciprocal social interaction test, 3-chamber sociability test, Porsolt forced swim test, acoustic startle response/prepulse inhibition test, and Barnes maze test. All the behavioral tests were carried out with 23 $Ptprd^{+/dISE}$ and 21 their WT littermates, 23 $Ptprd^{+/-}$ and 21 their WT littermates, and 18 $Ptprd^{dISS/dISS}$ and 16 their WT littermates and the behavioral testing started at 9-11 weeks of age. All the behavioral testing was performed between 8:30 a.m. and 18:30 p.m. Prior to all experiments, mice were left undisturbed in or near the soundproof testing room for at least 30 min to allow acclimation. After each trial of experiment the apparatus was thoroughly cleaned with hypochlorous water to eliminate any scent to prevent giving a bias as olfactory cue to a next subject. All the behavioral testing except the light/dark test, 3-chamber social interaction test, and Barnes maze test was performed with illumination level of 100 lux. The detailed procedures for each behavioral testing are as follows.

General health and neurological screening: Health status including body weight, rectal temperature, and neuromuscular strength was examined at the first day of the series of the

behavioral testing. Neuromuscular strength was examined by the grip strength test and wire hang test as described (1). A grip strength meter (O'Hara & Co., Tokyo, Japan) was used to assess forelimb grip strength. Each mouse was tested three times and the greatest value measured was used for statistical analysis. A box (215 × 22 × 23 cm) with a wire mesh grid (10 × 10 cm) on its top (O'Hara & Co., Tokyo, Japan) was used for the wire hang test. Latency to fall was recorded with a 60s cutoff time.

Light/dark transition test: Light/dark transition test was conducted as previously reported (2). Mice were placed into the dark chamber and allowed to move freely in the light (380 ± 20 lux) and dark chamber through the opening in between for 10 min. The total number of transitions between chambers, time spent in each side, latency to the first transmission to the light chamber, and distance traveled in each chamber were recorded.

Open field test: Locomotor activity was measured in an open field apparatus (40 × 40 × 30 cm; Accuscan Instruments, Columbus, OH, USA) as described (3). Mice were placed into left corner of the apparatus and allowed to move freely for 30 min. Total distance traveled, vertical activity (rearing measured by counting the number of photobeam interruptions), time spent in the center (20 × 20 cm) of the open field area, and the stereotypic counts were recorded using VersaMax system (Accuscan Instruments, Columbus, OH, USA).

Elevated pulse maze test: The elevated plus maze test was conducted as described (4). The elevated plus maze (O'Hara & Co., Tokyo, Japan) consisted of four arms (25 × 5 cm) arranged in plus shape with the 5 × 5 cm square in the center. Two of the arms were closed with 15 cm high walls and other two were open without walls. The closed and open arms were alternately arranged in the plus maze and the maze was elevated to a height of 55 cm above from floor. Each mouse was placed on the center of the maze facing one of the closed arms. Mouse behavior was recorded during a 10 min test period. The time spent in the open and closed arm, the number of entries into the arms, and the distance traveled were recorded.

Hot plate test: The hot plate test was conducted as described (5). Mice were placed on a 55.0 ± 0.3°C hot plate (Columbus Instruments, Columbus, OH, USA), and latency to the first front-paw response (rubbing the paws) was recorded.

Acoustic startle response and prepulse inhibition tests: The acoustic startle responses and prepulse inhibition tests were conducted using startle reflex measurement system (O'Hara & Co., Tokyo, Japan) as described (3). The subject mouse was put in the test equipment, a Plexiglas cylinder and the cylinder was placed in the test apparatus with background noise level at 70 dB for 10 min for habituation. A test session consisted of 6 trial types: two types for startle stimulus only trials, and four types for prepulse inhibition trials. The duration of white noise that was used as the startle stimulus was 40 ms for all trial types. The startle response was recorded for 140 ms (measuring the response every 1 ms) starting with the onset of the prepulse stimulus. The peak startle amplitude recorded during the 140 ms sampling window was used as the dependent variable. The intensity of startle stimulus was 110 or 120 dB. The prepulse sound was presented 100 ms before the startle stimulus, and its intensity was 74 or 78 dB. Four combinations of prepulse and startle stimuli were employed (74-110. 78-110, 74-120, and 78-120). Six blocks of the 6 trial types were presented in pseudorandom order such that each trial type was presented once within a block. The average intertrial interval was 15 s (range: 10–20 s). The startle amplitude and percentage of prepulse inhibition was measured.

Three-chamber sociability test: The test for sociability and preference for social novelty was conducted as previously described (6, 7). The apparatus comprised a rectangular, three-chambered box and a lid containing an infrared video camera (Ohara & Co.). Each chamber was 20 × 40 × 22 cm and the dividing walls were made from clear Plexiglas, with small square openings (5 × 3 cm) allowing access into each chamber. Before testing, the subject mice were individually placed in the middle chamber and allowed to freely explore the entire apparatus for 10 min for acclimatization. In the consecutive social preference test, an unfamiliar male mouse (stranger 1) that had no prior contact with the subject mouse was placed in one of the side chambers. The placement of stranger 1 in the left or right side chambers was systematically alternated between trials. The stranger mouse was enclosed in a small, circular wire cage that allowed nose contact between the bars but prevented fighting. The cage was 11 cm high, with a bottom diameter of 9 cm and bars spaced 0.5 cm apart. The subject mouse was first placed in the middle chamber and allowed to explore the entire social test box for 10 min. A second, unfamiliar mouse was placed in

the chamber that had been empty during the first 10-min session. The amount of time spent within a 5-cm distance of the wire cage and in each chamber, the number of entries into each chamber and each area within 5 cm of the wire cage, total distance traveled, and average speed were recorded using Image J 1.47a software (NIH). In the next 10-min social novelty preference test, second stranger was enclosed in an identical small wire cage. The test mouse had a choice between the already-investigated familiar mouse (stranger 1) in the social preference test, and the novel unfamiliar mouse (stranger 2). As described above, the social behaviors were measured and recorded. The stranger mice used in this experiment were 8 to 12-week-old C57BL/6J male mice. Analysis was performed automatically using Image CSI software.

Social interaction test in a novel environment: Social interaction test was conducted as previously described (8, 9). Two mice of identical genotypes that had not met before, were placed into a box together (40 cm × 40 cm × 30 cm) and allowed to explore freely for 10 min. The social behavior was monitored by a CCD camera, then, analysis was performed automatically using Image SI software. The total duration of contacts, the number of contacts, the total duration of active contacts, mean duration per contact, and total distance traveled were measured.

Porsolt forced swim test: Porsolt forced swim test was performed as previously described (7, 8). The apparatus consisted of a Plexiglas cylinder (20 cm high × 10 cm diameter) filled with water (23°C) up to a height of 7.5 cm. Mice were placed into the cylinders, and the immobility and the distance traveled were recorded over a 10-min test period. Images were captured at one frame per second. Data acquisition and analysis were performed automatically, using Image J based original program ImagePS software.

Barnes maze test: The Barnes maze test was conducted on a white circular platform, 1.0 m in diameter, with 12 holes equally placed around the perimeter (O'Hara & Co., Tokyo, Japan) elevated 75 cm from the floor as previously described (9). The platform was illuminated with 1200 lx lighting and one of the 12 holes leads to a black Plexiglas escape box (17 × 13 × 7 cm), being assigned to each subject as a target hole. Location of the target hole on the platform was evenly assigned to WT and mutant mice. Prior to the test, the subject had habituation trial to become familiar with the maze and the escape box. Each trial test started with emergence of the subject on the center of

the platform and ended when the subject entered the escape box through their assigned target hole or 5 min elapsed. The latency and distance for the subject to reach the target hole and the number of times that the subject visited incorrect holes were measured in all the tests. One to three trials per day were conducted as a training session until the number of incorrect visiting reached 1-2 on average. Twenty-four hrs after the training session, the mice were subjected to probe trial test (PT1) that measured the time spent around each hole during 3 min session without the escape box. Mice were left undisturbed until receiving next probe trials. Thirty days after the PT1, the mice were once again subjected to a probe trial test (PT2) to check remote memory. The time spent around each hole was recorded using Image BM software.

Figure S1

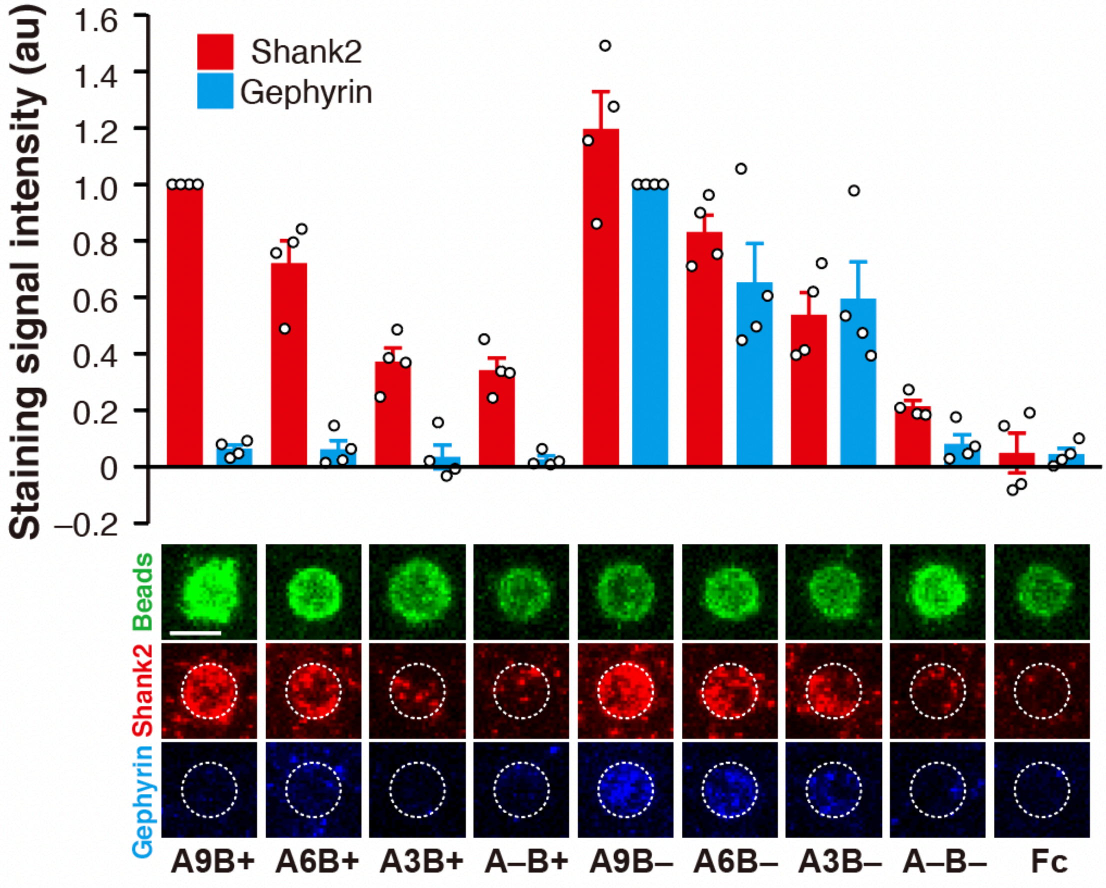

Fig. S1. Synaptogenic properties of PTPRD splice variants in cultured cerebral cortical neurons. Cocultures of beads conjugated with ECDs of PTPRD splice variants (green) and cortical neurons were immunostained with anti-Shank2 (red) and anti-gephyrin (blue) antibodies (bottom). Shank2 (red bars) and gephyrin (blue bars) staining signals on the beads were quantified (top). Scale bar, 5 μm. The dashed circles indicate the positions of the beads. Data are presented as mean ± s.e.m. (n = 4 experiments).

Figure S2

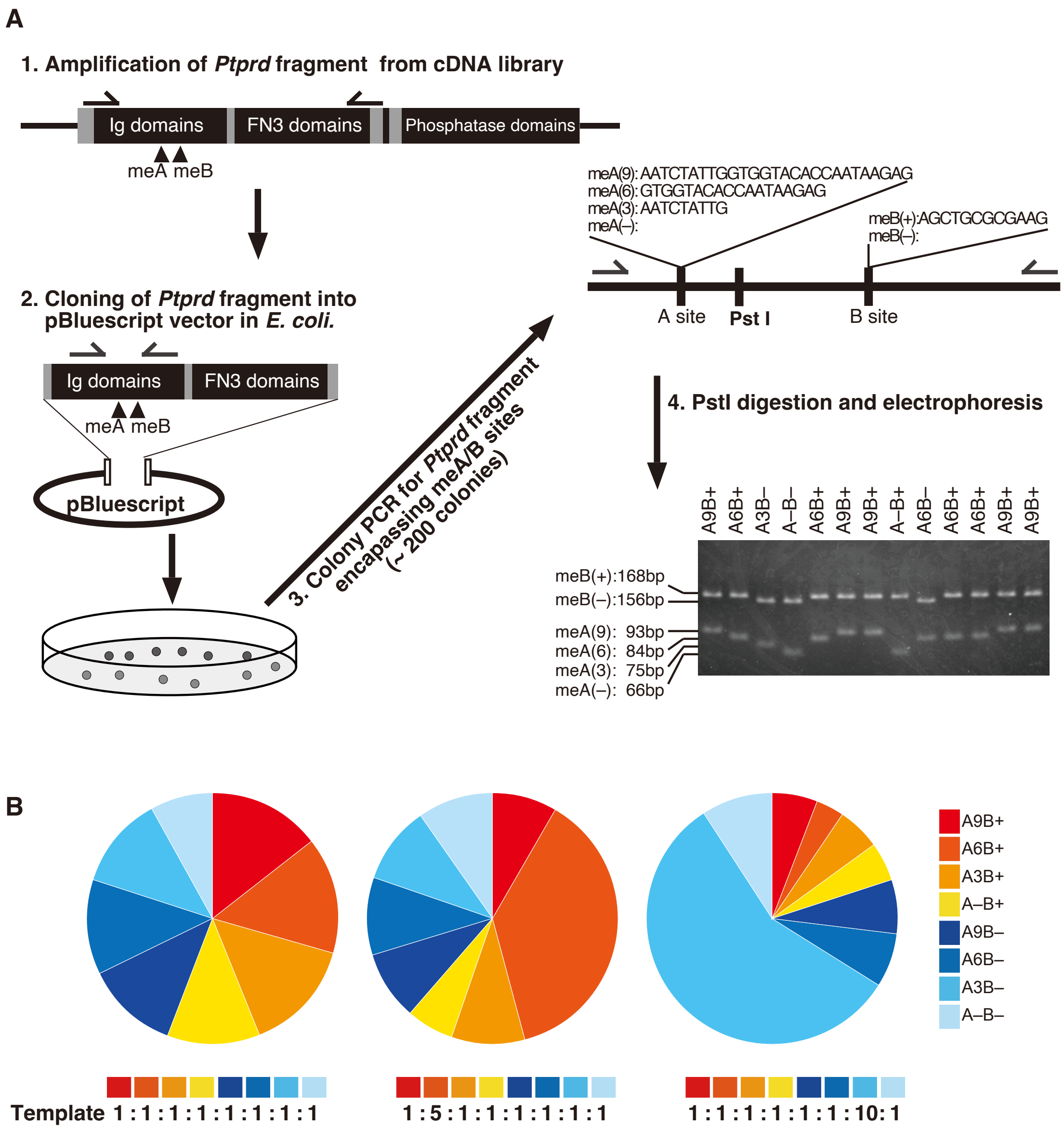

Fig. S2. Flow diagram of *Ptprd* cDNA fingerprinting.

(A) Flow diagram of *Ptprd* cDNA fingerprinting to profile eight *Ptprd* splice variants by inclusion or exclusion of meA3, meA6, and meB. (B) Validation of *Ptprd* cDNA fingerprinting. The pie charts show the results of cDNA fingerprinting using the eight *Ptprd* splice variants with known abundances shown below as templates.

Figure S3

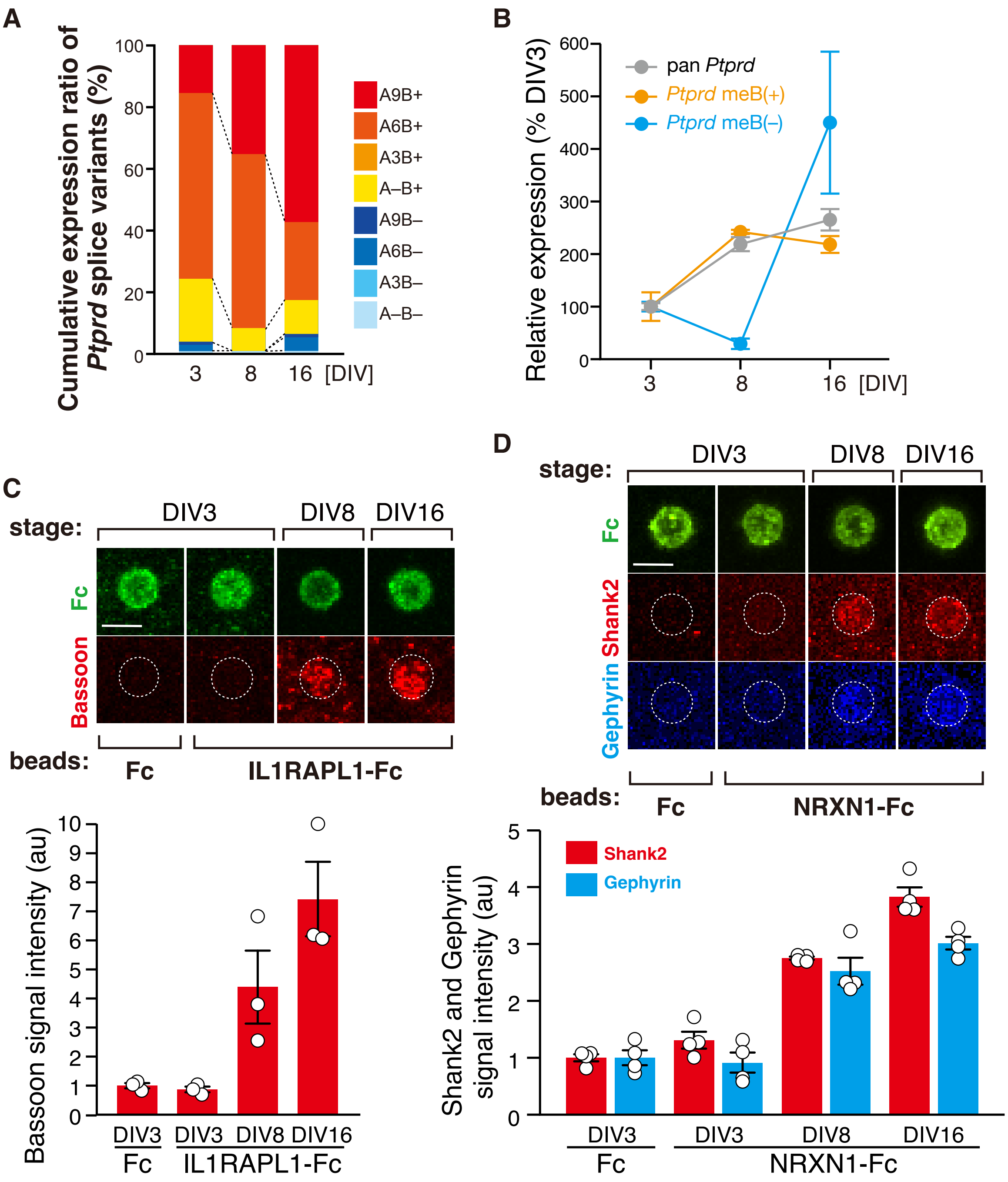

Fig. S3. Developmental changes of *Ptprd* splice variants' expression and synaptogenic activity of cerebral cortical neuronal cultures.

(A) Relative abundance ratios of eight *Ptprd* splice variants in the cultured cerebral cortical neurons at days in vitro (DIV) 3, 8, and 16 (n = 3). (B) Expression levels of total *Ptprd* (gray), meB-containing

Ptprd variants (orange), and meB-lacking *Ptprd* variants (blue) in cerebral cortical neurons at DIV 3, 8, and 16 are estimated by real-time PCR (n = 3 – 12 experiments). (C) Presynapse-inducing activity of cerebral cortical neurons at DIV 3, 8, and 16 estimated by hemi-synapse formation assay with IL1RAPL1-Fc beads. Representative images (top) and quantitative measurements (bottom) of excitatory presynaptic differentiation visualized by Bassoon signal accumulation on IL1RAPL1-Fc beads (n = 3 experiments). (D) Postsynapse-inducing activity of cerebral cortical neurons at DIV 3, 8, and 16 estimated by hemi-synapse formation assay with NRXN1-Fc beads. Representative images (top) and quantitative measurements (bottom) of excitatory and inhibitory postsynaptic differentiation visualized by Shank2 and Gephyrin signal accumulation on NRXN1-Fc beads, respectively (n = 4 experiments). Data are presented as mean ± s.e.m.

Figure S4

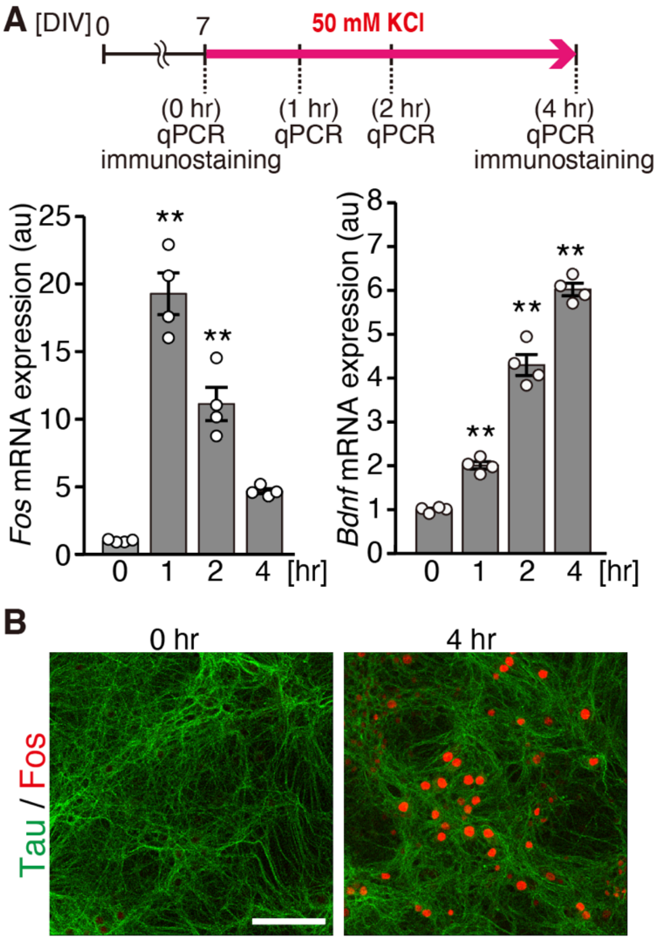

Fig. S4. Induction of *Fos* and *Bdnf* expressions by KCl stimulation.

(A) Experimental design (top), and expression levels of *Fos* (Bottom left) and *Bdnf* (Bottom right) estimated by real-time PCR (n = 4 each time point). Data are mean ± s.e.m. **p < 0.01, Dunnett's test. (B) Representative immunostaining images for Tau (green) and Fos (red) after 0 (left) and 4 (right) hours of KCl treatment. Scale bar, 100 µm.

Figure S5

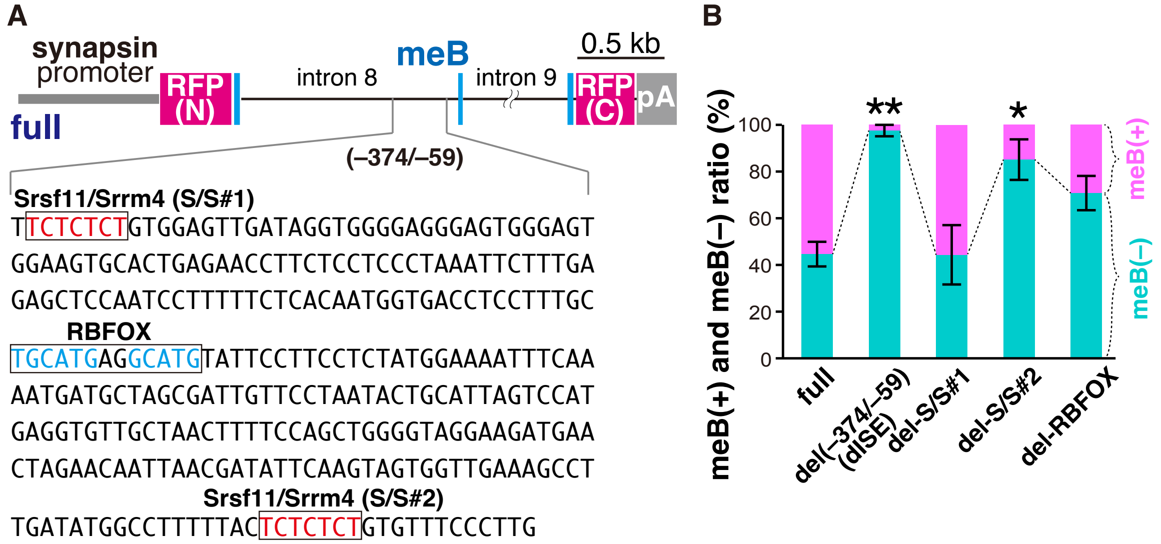

Fig. S5. Dissection of intronic splicing enhancer region for *Ptprd* meB.

(A) Schematic presentation of reporter mini-genes to screen intronic splicing enhancer element for meB. The sequence of the 316 bp ISE region and the putative splicing factor binding sequences are shown. The boxed sequences were deleted in the mutants used in the experiments. (B) The RFP cDNA derived from the mini-reporter gene was cloned and meB inclusion/exclusion rates were examined by PCR (n = 4 experiments). Data are mean ± s.e.m. *p < 0.05 and **p < 0.01, Dunnett's test.

Figure S6

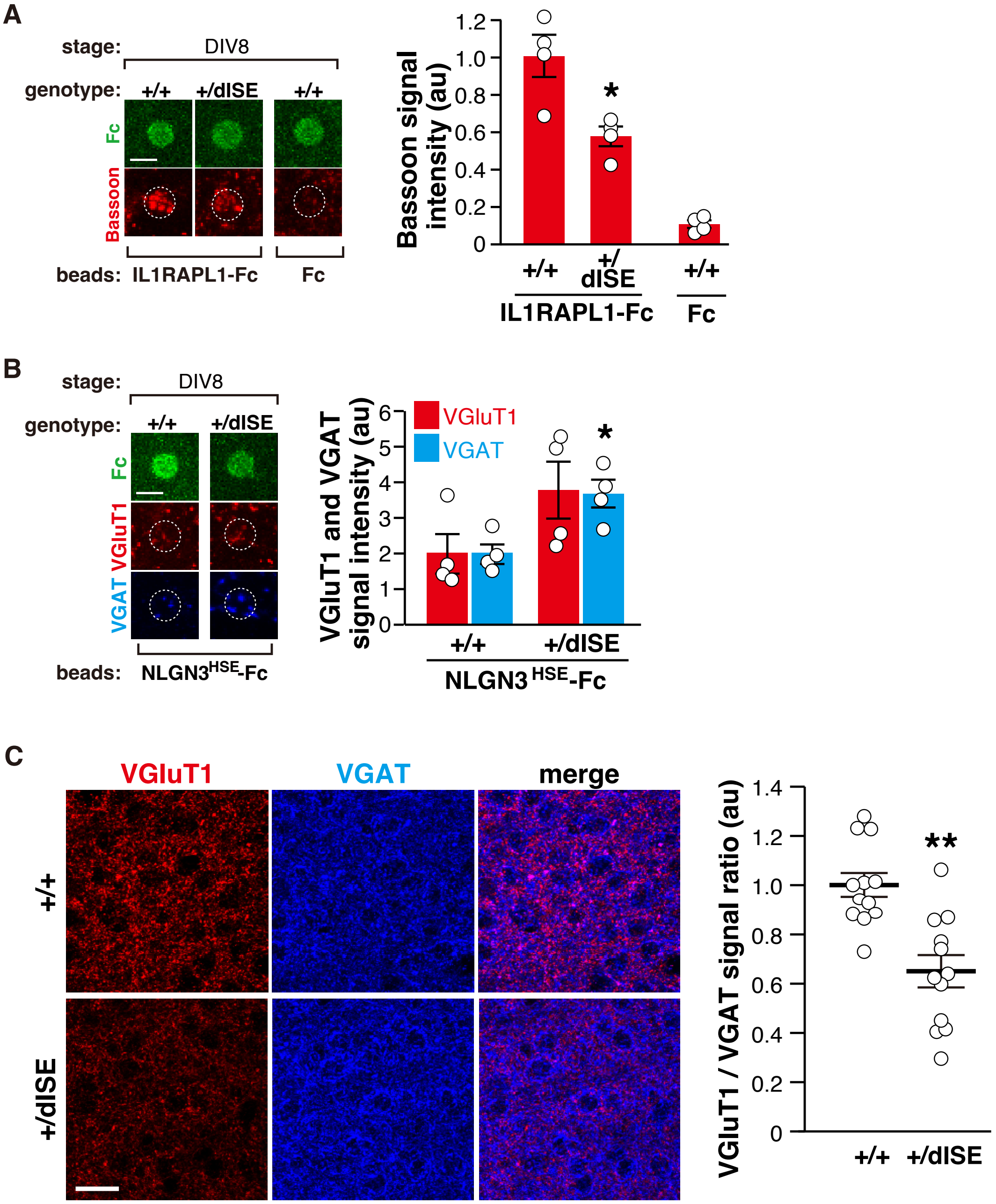

Fig. S6. Imbalance in excitatory and inhibitory synapses in *Ptprd*$^{+/dISE}$ mice.

(A) Decrease in PTPRD meB(+) variants-mediated excitatory synaptogenic activity in primary cerebral cortical neurons from *Ptprd*$^{+/dISE}$ mice. Representative images (left) and quantitative measurements (right) of excitatory presynaptic differentiation visualized by Bassoon signal accumulation on IL1RAPL1-Fc beads (n = 4 experiments). (B) Increase in PTPRD meB(–) variants-mediated inhibitory synaptogenic activity in primary cerebral cortical neurons from *Ptprd*$^{+/dISE}$ mice.

Representative images (left) and quantitative measurements (right) of excitatory and inhibitory presynaptic differentiation on NLGN3^{HSE}-Fc beads. Excitatory and inhibitory presynaptic terminals were visualized by immunostaining for VGluT1 (red) and VGAT (blue), respectively (n = 4 experiments). The dashed circles indicate the positions of the beads. (C) Immunohistochemistry for VGluT1 and VGAT in somatosensory cortex of wild-type (+/+) and littermate heterozygous mutant (+/dISE) mice. Ratios of staining signal intensity for VGAT and VGluT1 are quantified on the right (n = 12 slices from 2 mice each). Scale bars, 5 μm in (A and B) and 20μm in (C). Data are mean ± s.e.m. *p < 0.05 and **p < 0.01, Two-sided Student's t-test.

Figure S7

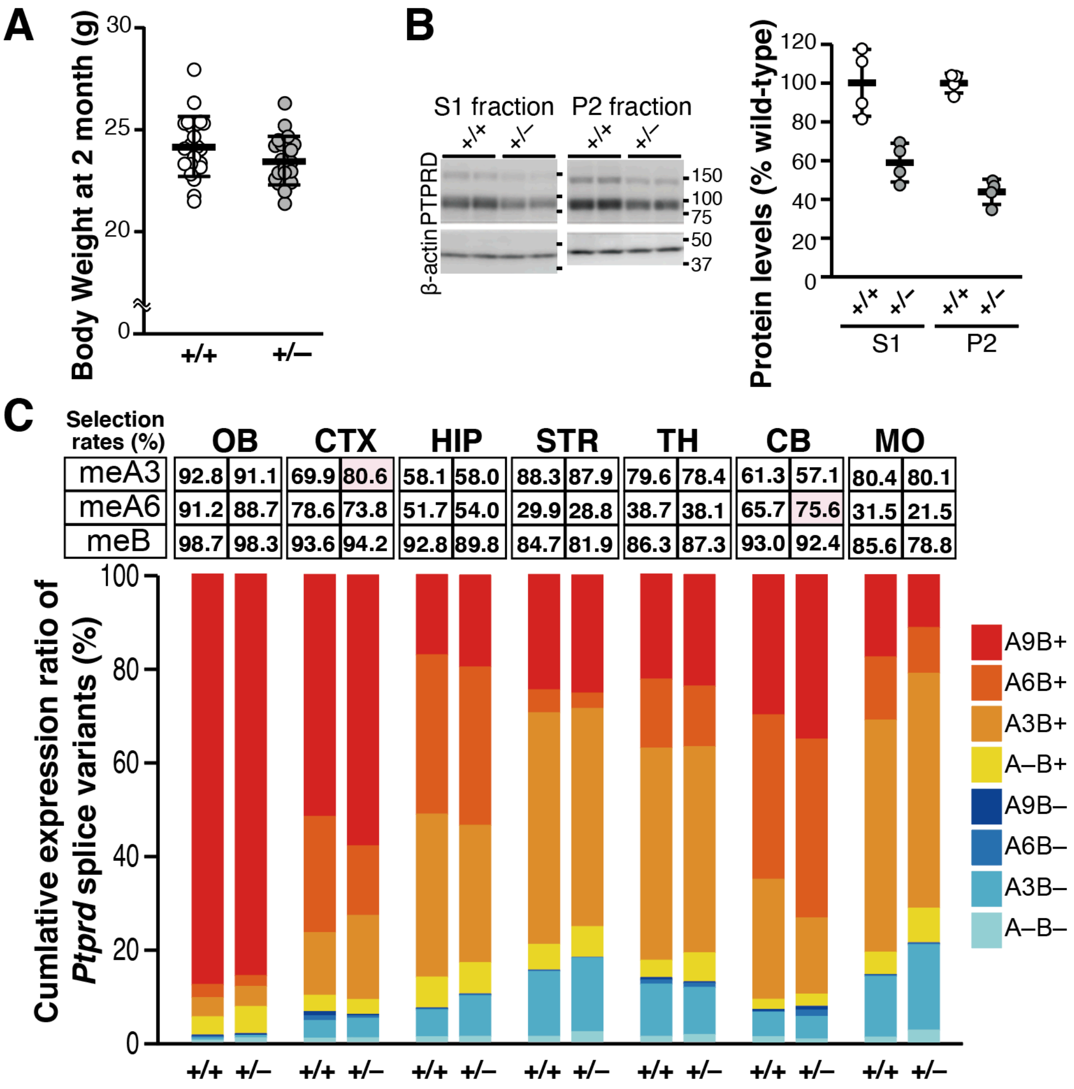

Fig. S7. meA/B profiling of heterozygous *Ptprd* knockout mice.

(A) Body weights of 8-week-old heterozygous *Ptprd* knockout mice (N=23) and their WT littermates (N = 21). (B) Representative immunoblots (left) and quantification of expression levels (right) of total PTPRD protein in the whole brain S1 and synaptosomal P2 fraction in *Ptprd*$^{+/-}$ mice and their WT littermates (N = 4 each). (C) Selection rates of meA3, meA6 and meB (top) and relative abundance ratios of eight *Ptprd* splice variants (bottom) in the olfactory bulb (OB), cerebral cortex (CTX), hippocampus (HIP), striatum (STR), thalamus (TH), cerebellum (CB), and medulla oblongata (MO) of 8-week-old wild-type (*Ptprd*$^{+/+}$) and heterozygous knockout (*Ptprd*$^{+/-}$) mice (N = 4 each). Statistically significant differences in two-tailed t-test are shaded in pink. Data are mean ± s.d..

Figure S8

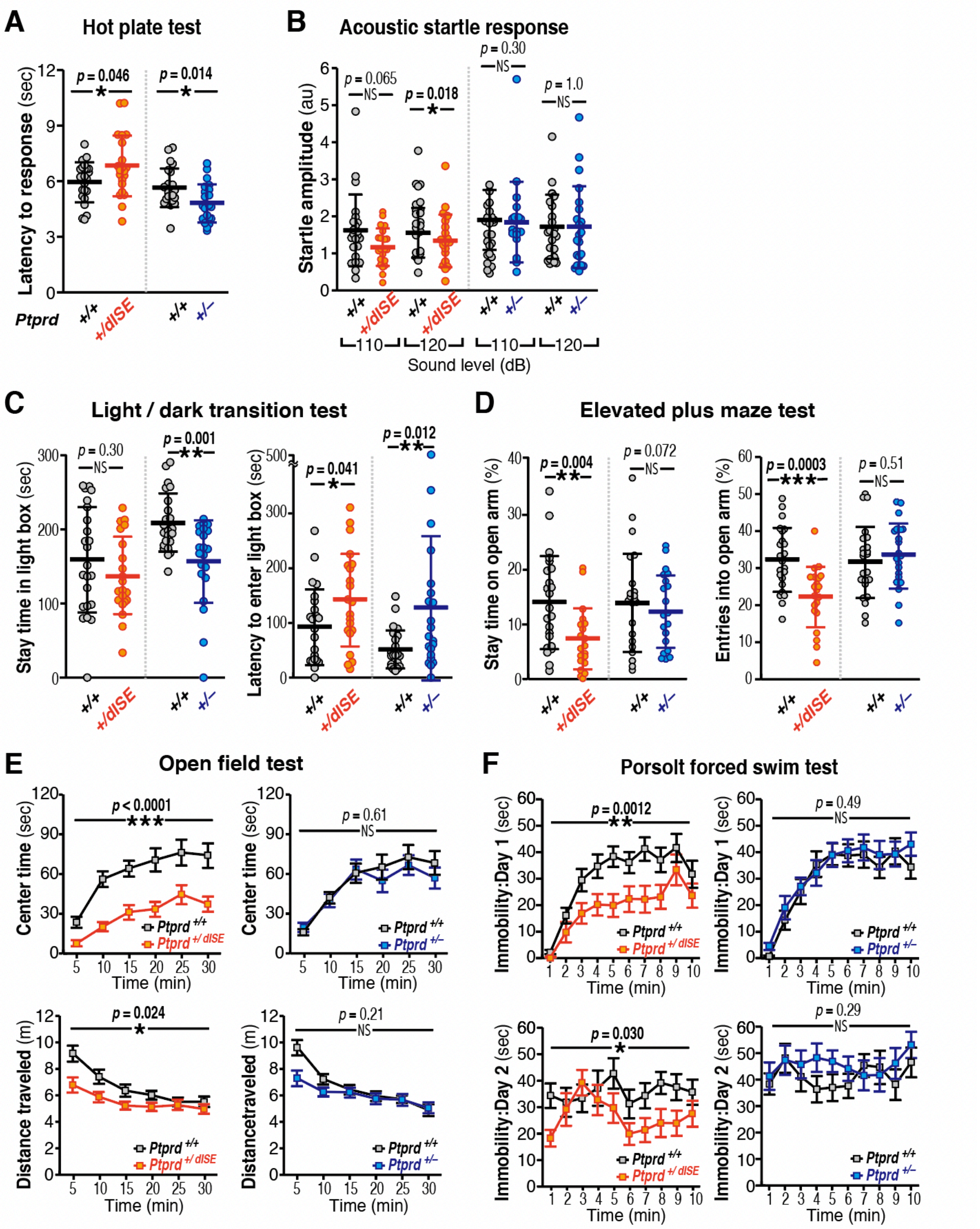

Fig. S8. Pain and auditory-related sensory responses, anxiety-related behaviors, and anti-depression-like behaviors in heterozygous dISE mutant (*Ptprd*$^{+/dISE}$) and heterozygous knockout (*Ptprd*$^{+/–}$) mice.

(A and B) Hot plate latency (A) and acoustic startle responses (B) were measured. (C to E) Anxiety-related behaviors evaluated by light/dark transition test (C), elevated plus maze test (D), and open field test (E). (F) Immobile time in forced swim test on two consecutive days were measured. *$p < 0.05$, **$p < 0.01$, and ***$p < 0.001$, Two-tailed t-test. Data are mean ± s.d. in (A) to (D) and mean ± s.e.m. in (E) and (F). N = 23 and 21 for *Ptprd*$^{+/dISE}$ mice and WT littermates, and N = 23 and 21 for *Ptprd*$^{+/–}$ mice and WT littermates, respectively.

Figure S9

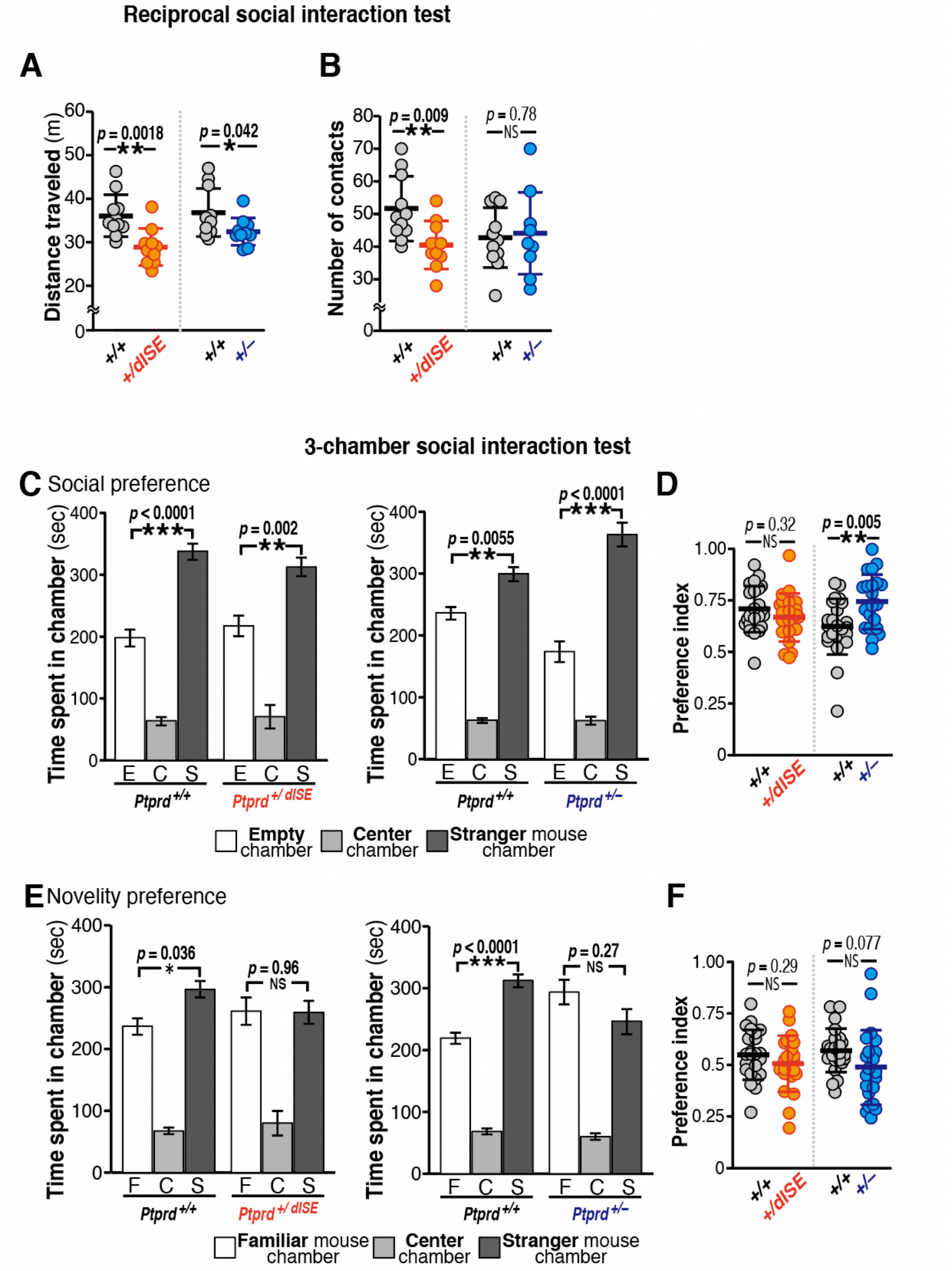

Reciprocal social interaction test
A
B
Distance traveled (m)
p = 0.0018
**
p = 0.042
*
+/+
+/dISE
+/+
+/–
Number of contacts
p = 0.009
**
p = 0.78
NS
3-chamber social interaction test
C Social preference
D
Time spent in chamber (sec)
p < 0.0001
***
p = 0.002
**
p = 0.0055
**
p < 0.0001
***
E C S
Ptprd +/+
Ptprd +/dISE
Ptprd +/–
Empty chamber
Center chamber
Stranger mouse chamber
Preference index
p = 0.32
NS
p = 0.005
**
E Novelty preference
F
p = 0.036
*
p = 0.96
NS
p < 0.0001
***
p = 0.27
NS
F C S
p = 0.29
NS
p = 0.077
NS
Familiar mouse chamber
Center chamber
Stranger mouse chamber

Fig. S9. Social behaviors in heterozygous dISE mutant (*Ptprd*$^{+/dISE}$) and heterozygous knockout (*Ptprd*$^{+/–}$) mice.

(A and B) Reciprocal social interaction tests of *Ptprd*$^{+/dISE}$ and *Ptprd*$^{+/–}$ mice (N = 11 and 10 pairs for *Ptprd*$^{+/dISE}$ mutant vs. mutant and littermate WT vs. WT, respectively, and N = 11 and 10 pairs for *Ptprd*$^{+/–}$ mutant vs. mutant and littermate WT vs. WT, respectively). Travel distance (A) and number of contacts (B) during 10 minutes test were measured. (C to F) Three-chamber sociability test of *Ptprd*$^{+/dISE}$ mice (N = 23 and 21 for mutant and WT mice, respectively) and *Ptprd*$^{+/–}$ mice (N = 23 and 21 for mutant and WT mice, respectively) to evaluate social preference (C and D) and novelty preference (E and F). Time spent in each chamber (C and E) and preference index (((time spent around stranger cage/(Time spent around stranger cage + Time spent around empty or familiar cage)) × 100) – 50) (D and F) is presented. Data are mean ± s.d. in (A), (B), (D), and (F), and mean ± s.e.m. in (C) and (E). *p < 0.05, **p < 0.01, and ***p < 0.001, Student's t-test in (A), (B), and (D), and paired t-test in (C) and (E).

Figure S10

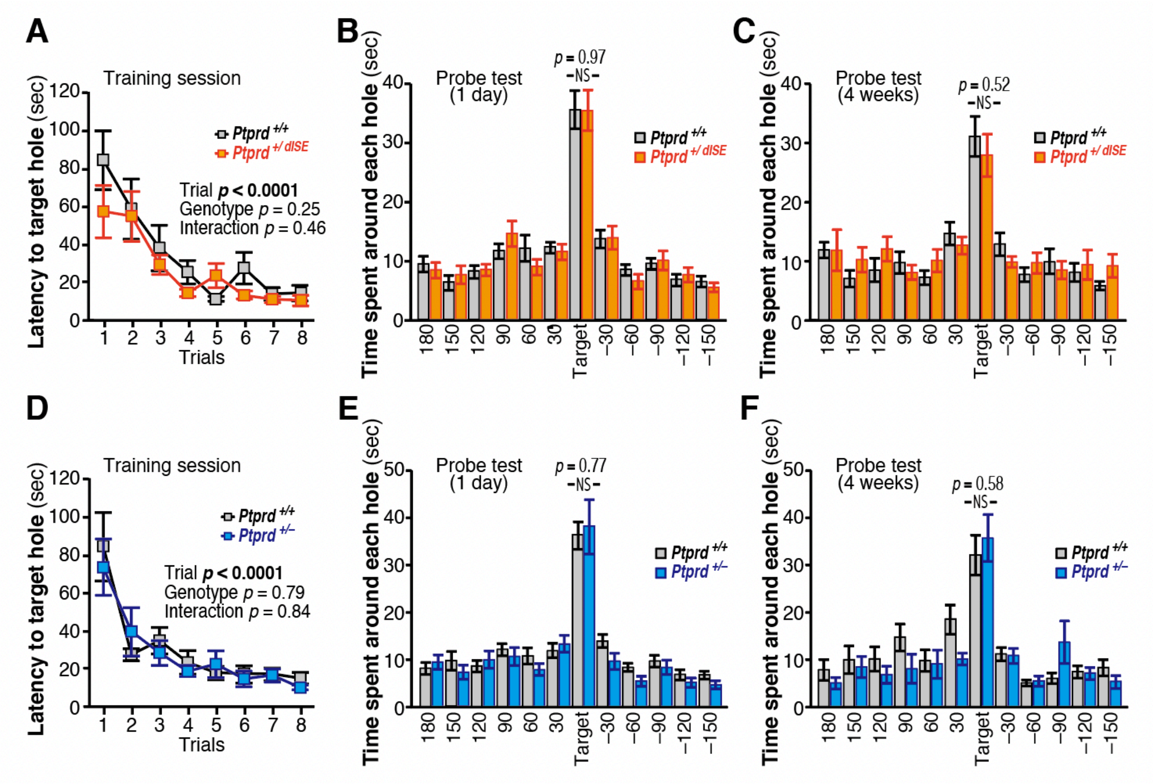

Fig. S10. Spatial learning and memory in heterozygous dISE mutant (*Ptprd*$^{+/dISE}$) and heterozygous knockout (*Ptprd*$^{+/–}$) mice.

(A to F) Spatial reference memory of *Ptprd*$^{+/dISE}$ mutants (N = 23) and WT littermates (N = 21) (A to C), and that of the *Ptprd*$^{+/–}$ mutants (N = 23) and WT littermates (N = 21) (D to F) were examined using the Barnes maze test. Latency to reach the target hole across training (A and D), time spent around each hole in the probe trial conducted 1 day (B and E) and 4 weeks (C and F) after last training session. All values are presented as the mean ± s.e.m.

Figure S11

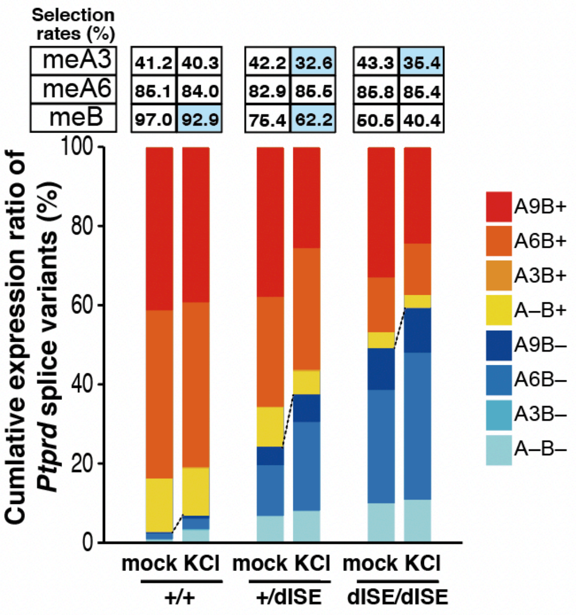

Fig. S11. meA/B profiling of cerebral cortical neurons from WT, *Ptprd*$^{+/dISE}$, and *Ptprd*$^{dISE/dISE}$ mice. Selection rates of meA3, meA6 and meB (top) and relative abundance ratios of eight *Ptprd* splice variants (bottom) in cultured neurons with or without KCl stimulation (N = 2, 3, and 3 for *Ptprd*$^{+/+}$, *Ptprd*$^{+/dISE}$, and *Ptprd*$^{dISE/dISE}$ mice, respectively). Statistically significant differences in two-tailed t-test are shaded in light blue.

Figure S12

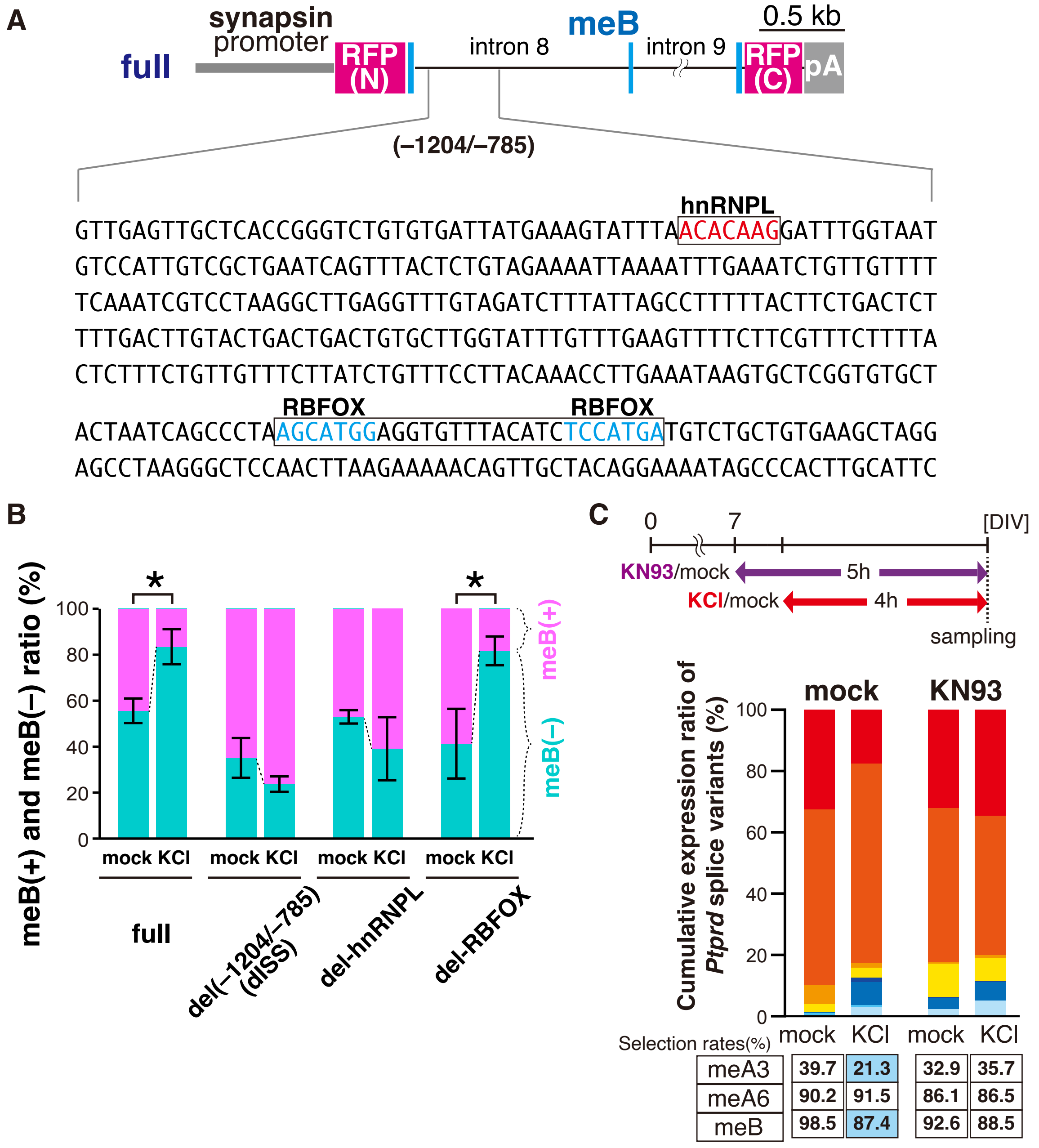

Fig. S12. Dissection of intronic splicing silencer region for *Ptprd* meB.

(A) Schematic presentation of reporter mini-genes to screen neuronal activity-dependent intronic splicing silencer element for meB. The sequence of the 420 bp ISS region and the putative splicing factor binding sequences are shown. The boxed sequences were deleted in the mutants used in the experiments. (B) The RFP cDNA derived from the mini-reporter gene was cloned and meB

inclusion/exclusion rates were examined (n = 4 experiments). Data are mean ± s.e.m. *$p < 0.05$, Two-sided Student's t-test. (C) Blockade of neuronal activity-dependent meB skipping by CaMKII/CaMKIV inhibitor KN93. Experimental design (top) and relative abundance ratios of eight *Ptprd* splice variants and selection rates of meA3, meA6, and meB (bottom) in the mock- and KN93-treated primary cerebral cortical neurons (n = 5–6 experiments). Statistically significant differences in two-tailed t-test are shaded in light blue.

Figure S13

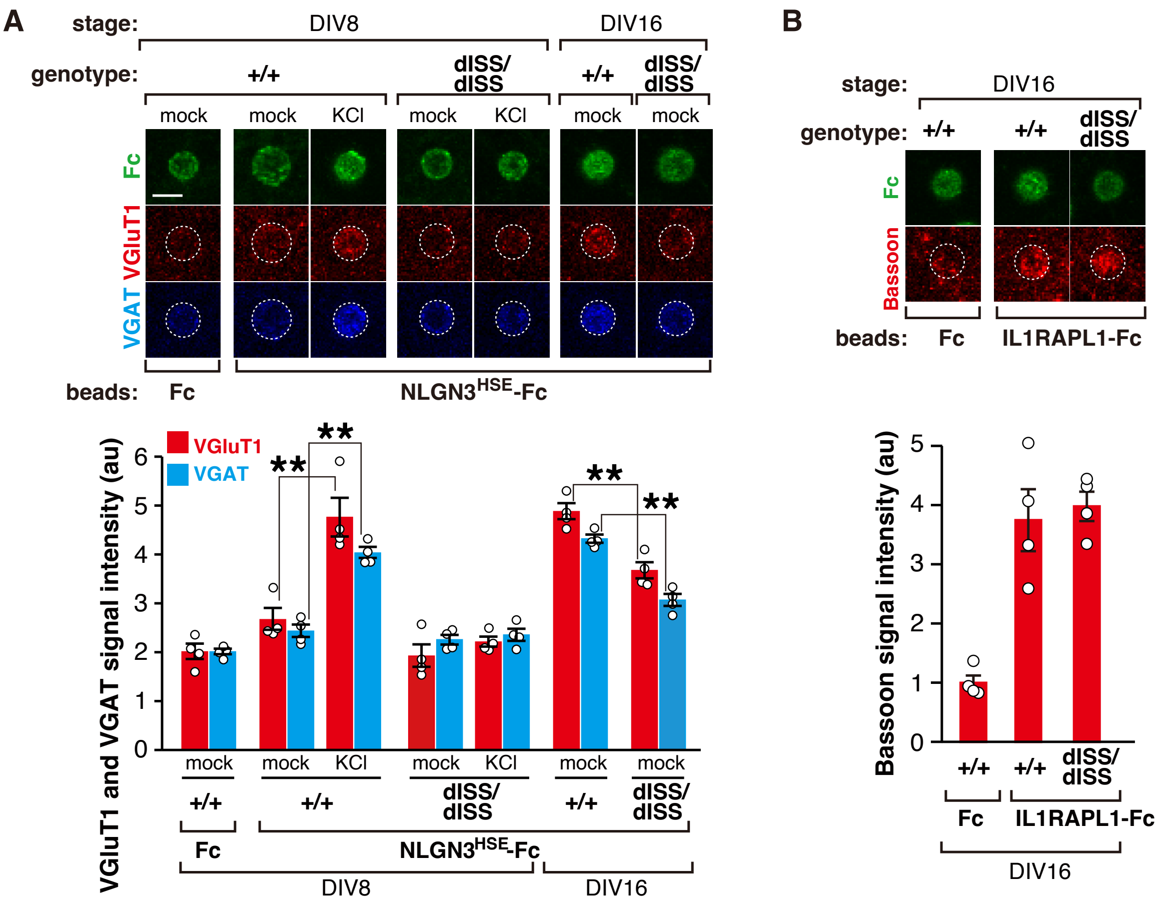

Fig. S13. Impairments in KCl-induced PTPRD meB(–) variants-mediated synaptogesis in *Ptprd*$^{dISS/dISS}$ neurons.

(A) PTPRD meB(–) variants-mediated excitatory and inhibitory synaptogenic activity in primary cerebral cortical neurons from *Ptprd*$^{dISS/dISS}$ mice. Representative images (top) and quantitative measurements (bottom) of excitatory and inhibitory presynaptic differentiation on NLGN3^{HSE}-Fc beads at DIV8 and 16. Excitatory and inhibitory presynaptic terminals were visualized by immunostaining for VGluT1 (red) and VGAT (blue), respectively (n = 4 experiments). Experimental design of KCl treatment is same as Figure 1I. The dashed circles indicate the positions of the beads.

(B) PTPRD meB(+) variants-mediated excitatory synaptogenic activity in primary cerebral cortical neurons from *Ptprd*$^{dISS/dISS}$ mice. Representative images (top) and quantitative measurements (bottom) of excitatory presynaptic differentiation visualized by Bassoon signal accumulation on

IL1RAPL1-Fc beads at DIV16 (n = 4 experiments). Scale bars, 5 μm. Data are mean ± s.e.m. $^{*}p < 0.05$ and $^{**}p < 0.01$, Two-sided Student's t-test.

Figure S14

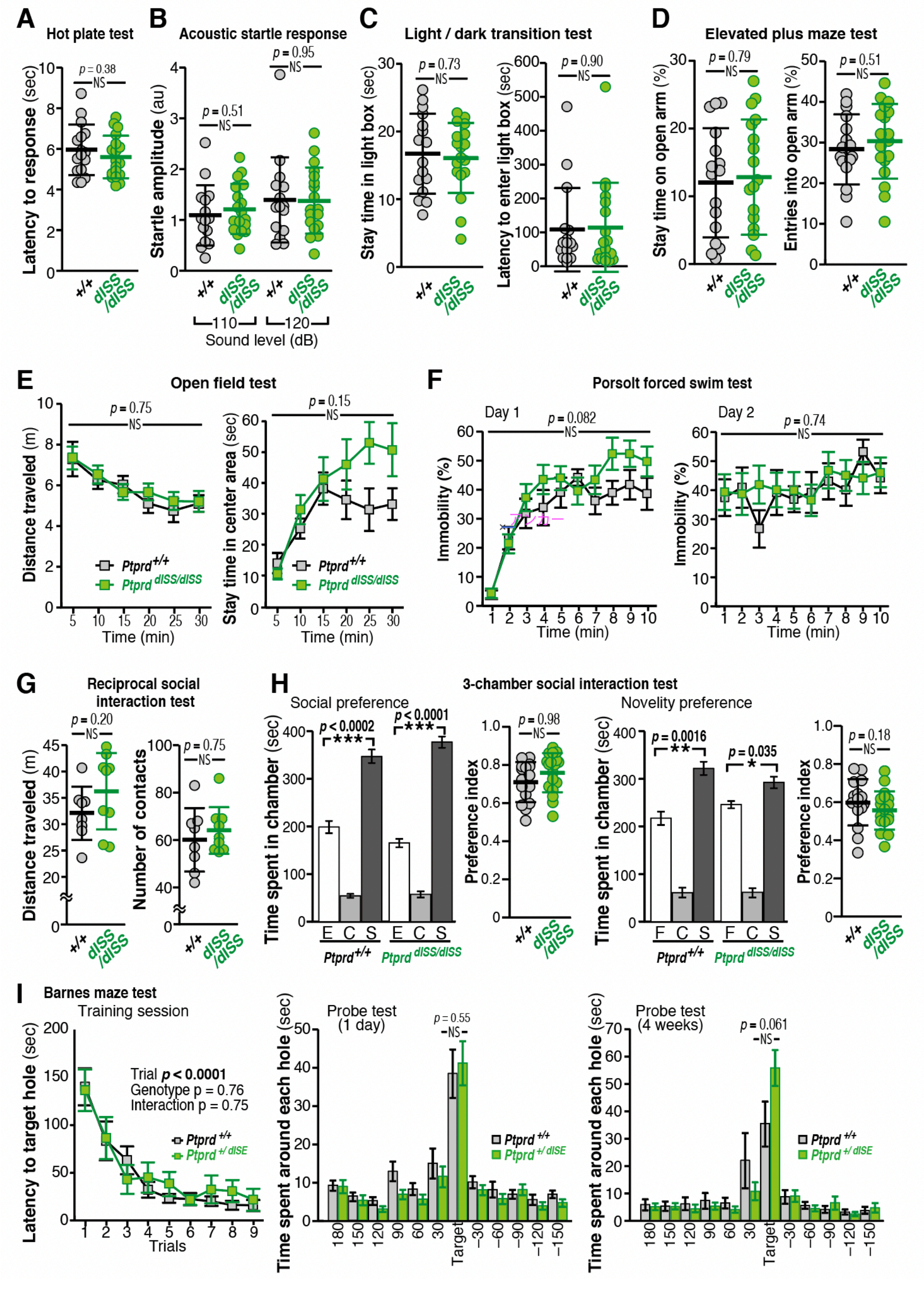

Fig. S14. Behavioral test battery of *Ptprd*$^{dISS/dISS}$ mutant mice (N = 18) and WT littermates (N = 16).

(A) Hot plate latency. (B) Acoustic startle responses. (C) Stay time in light box (left) and latency to enter light box (right) in light/dark transition test. (D) Stay time on open arm (left) and number of entries into open arm (right) in elevated plus maze test. (E) Travel distance (left) and stay time in center area (right) in open field test. (F) Immobile time in Porsolt forced swim test on two consecutive days. (G) Travel distance (left) and number of contacts (right) during reciprocal social interaction tests (N = 9 and 8 pairs for *Ptprd*$^{dISS/dISS}$ mutant vs. mutant and littermate WT vs. WT, respectively). (H) Three-chamber sociability test to evaluate social preference (left) and novelty preference (right). Time spent in each chamber and preference index (((time spent around stranger cage/(Time spent around stranger cage + Time spent around empty or familiar cage)) × 100) – 50) are presented. (I) Barnes maze test. Latency to reach the target hole across training (left), time spent around each hole in the probe trial conducted 1 day (middle) and 4 weeks (right) after last training session. Values are presented as the mean ± s.d. in (A) to (D) and (G), and preference indices in (H), and as the mean ± s.e.m. in (E) and (F), time spent in chamber in (H), and (I).

Table S1. Results of behavioral test battery on *Ptprd*$^{dISS/dISS}$ mice

	Behavioral tests	*Ptprd*$^{dISS/dISS}$ vs WT
Sense modality	Hot plate test	Hot plate latency
		Comparable to WT
	Acoustic startle response	Startle amplitude
		Comparable to WT
Motor coordination/ learning	Rota-rod test	Motor coordination
		Comparable to WT
		Motor learning
		decreased*
Locomotor activity	Open field test	General locomotor activity
		Comparable to WT
Anxiety	Light & Dark transition test	Total distance traveled
		Comparable to WT
		Latency to light area
		Comparable to WT
	Elevated plus maze	Stay time on open arms
		Comparable to WT
Sensory motor gating	Prepulse inhibition test	Prepulse inhibition
		Comparable to WT
Sociability	Reciprocal social test	Total contacts
		Comparable to WT
	3-chamber sociability test	Social preference
		Comparable to WT
		Novelty preference
		Comparable to WT
Depression	Porsolt forced swim test	Immobilized time
		Comparable to WT
		Distance swum
		Comparable to WT

SI References

1. M. Inui, M. Miyado, M. Igarashi, M. Tamano, A. Kubo, S. Yamashita, H. Asahara, M. Fukami, S. Takada, Rapid generation of mouse models with defined point mutations by the CRISPR/Cas9 system. *Sci. Rep*. **4**, 5396 (2014).

2. M. Hashimoto, T. Takemoto, Electroporation enables the efficient mRNA delivery into the mouse zygotes and facilitates CRISPR/Cas9-based genome editing. *Sci. Rep.* **5**, 11315 (2015).

3. K. Yoshimi, Y. Oka, Y. Miyasaka, Y. Kotani, M. Yasumura, Y. Uno, K. Hattori, A. Tanigawa, M. Sato, M. Oya, K. Nakamura, N. Matsushita, K. Kobayashi, T. Mashimo, Combi-CRISPR: combination of NHEJ and HDR provides efficient and precise plasmid-based knock-ins in mice and rats. *Hum. Genet.* **140**, 277–287 (2021).

4. T. Yoshida, M. Yasumura, T. Uemura, S. Lee, M. Ra, R. Taguchi, Y. Iwakura, M. Mishina, IL-1 receptor accessory protein-like 1 associated with mental retardation and autism mediates synapse formation by trans-synaptic interaction with protein tyrosine phosphatase δ. *J. Neurosci.* **38**, 13485–13499 (2011).

5. T. Uemura, S. Lee, M. Yasumura, T. Takeuchi, T Yoshida, M. Ra, R. Taguchi, K. Sakimura, M. Mishina, Trans-Synaptic interaction of GluR δ2 and Neurexine through Cbl1 mediates synapse formation in the cerebellum. *Cell* **141**, 1068-1079 (2010).

6. T. Yoshida, A. Yamagata, A. Imai, J. Kim, H. Izumi, S. Nakashima, T. Shiroshima, A. Maeda, S. Iwasawa-Okamoto, K. Azechi, F. Osaka, T. Saitoh, K. Maenaka, T. Shimada, Y. Fukata, M. Fukata, J. Matsumoto, H. Nishijo, K. Takao, S. Tanaka, S. Okabe, K. Tabuchi, T. Uemura, M. Mishina, H. Mori, S. Fukai, Canonical versus non-canonical transsynaptic signaling of neuroligin 3 tunes development of sociality in mice. *Nat. Commun.* **12**, 1848 (2021).

7. J. R. Arron, M. M. Winslow, A. Polleri, C. Chang, H. Wu, X. Gao, J. R. Neilson, L. Chen, J. J. H, S. K. Kim, N. Yamasaki, T. Miyakawa, U. Francke, I. A. Graef, G. R. Crabtree, NFAT dysregulation by increased dosage of DSCR1 and DYRK1A on chromosome 21. *Nature* **441**, 595–600 (2006).

8. K. Takao, T. Miyakawa, Light/dark transition test for mice. *J. Vis. Exp.* **1**, e104 (2006).

9. M. Ihara, N. Yamasaki, A. Hagiwara, A. Tanigaki, A. Kitano, R. Hikawa, H. Tomimoto, M. Noda, M. Takanashi, H. Mori, N. Hattori, T. Miyakawa, M. Kinoshita, Sept4, a component of presynaptic scaffold and Lewy bodies, is required for the suppression of a-synuclein neurotoxicity. Neuron 53, 519–533 (2007).

10. M. Komada, K. Takao, T. Miyakawa, Elevated plus maze for mice. *J. Vis. Exp.* **22**, e1088 (2008).

11. T. Miyakawa, E. Yared, J. H. Pak, F. L. Huang, K. Huang, J. N. Crawley, Neurogranin null mutant mice display performance deficits on spatial learning tasks with anxiety related components. *Hippocampus* **11**, 763–775 (2001).

12. S. S. Moy, J. J. Nadler, A. Perez, R. P. Barbaro, J. M. Johns, T. R. Magnuson, J. Piven, J. N. Crawley, Sociability and preference for social novelty in five inbred strains: an approach to assess autistic-like behavior in mice. *Genes Brain Behav.* **3**, 287–302 (2004).

13. K. Tanda, A. Nishi, N. Matsuo, K. Nakanishi, N. Yamasaki, T. Sugimoto, K. Toyama, K. Takao, T. Miyakawa, Abnormal social behavior, hyperactivity, impaired remote spatial memory, and increased D1-mediated dopaminergic signaling in neuronal nitric oxide synthase knockout mice. *Mol. Brain* **2**, 19 (2009)

14. N. Matsuo, T. Takao, K. Nakanishi, N. Yamasaki, K. Tanda, T. Miyakawa, Behavioral profiles of three C57BL/6 substrains. *Front. behav. neurosci.* **4**, 29 (2010).

15. T. Miyakawa, L. M. Leiter, D. J. Gerber, R. R. Gainetdinov, T. D. Sotnikova, H. Zeng, M. G. Caron, S. Tonegawa, Conditional calcineurin knockout mice exhibit multiple abnormal behaviors related to schizophrenia. *Proc. Natl. Acad. Sci. U.S.A.* **100**, 8987–8992 (2003).
